# Interruption of p38^MAPK^-MSK1-CREB-MITF-M pathway to prevent hyperpigmentation in the skin

**DOI:** 10.7150/ijbs.93120

**Published:** 2024-02-17

**Authors:** Song-Hee Kim, Jiyeon Lee, Jihye Jung, Ga Hyun Kim, Cheong-Yong Yun, Sang-Hun Jung, Bang Yeon Hwang, Jin Tae Hong, Sang-Bae Han, Jae-Kyung Jung, Youngsoo Kim

**Affiliations:** 1 College of Pharmacy, Chungbuk National University, Cheongju 28160, Korea.; 2 College of Pharmacy, Chungnam National University, Daejeon 34134, Korea.

**Keywords:** Melanin pigmentation, UV-B, α-MSH, MKK3, benzimidazole-2-butanol

## Abstract

**Background:** Melanocortin 1 receptor (MC1R), a receptor of α-melanocyte-stimulating hormone (α-MSH), is exclusively present in melanocytes where α-MSH/MC1R stimulate melanin pigmentation through microphthalmia-associated transcription factor M (MITF-M). Toll-like receptor 4 (TLR4), a receptor of endotoxin lipopolysaccharide (LPS), is distributed in immune and other cell types including melanocytes where LPS/TLR4 activate transcriptional activity of nuclear factor (NF)-κB to express cytokines in innate immunity. LPS/TLR4 also up-regulate MITF-M-target melanogenic genes in melanocytes. Here, we propose a molecular target of antimelanogenic activity through elucidating inhibitory mechanism on α-MSH-induced melanogenic programs by benzimidazole-2-butanol (BI2B), an inhibitor of LPS/TLR4-activated transcriptional activity of NF-κB.

**Methods:** Ultraviolet B (UV-B)-irradiated skins of HRM-2 hairless mice and α-MSH-activated melanocyte cultures were employed to examine melanogenic programs.

**Results:** Topical treatment with BI2B ameliorated UV-B-irradiated skin hyperpigmentation in mice. BI2B suppressed the protein or mRNA levels of melanogenic markers, such as tyrosinase (TYR), MITF-M and proopiomelanocortin (POMC), in UV-B-exposed and pigmented skin tissues. Moreover, BI2B inhibited melanin pigmentation in UV-B-irradiated co-cultures of keratinocyte and melanocyte cells and that in α-MSH-activated melanocyte cultures. Mechanistically, BI2B inhibited the activation of cAMP response element-binding protein (CREB) in α-MSH-induced melanogenic programs and suppressed the expression of MITF-M at the promoter level. As a molecular target, BI2B primarily inhibited mitogen-activated protein kinase (MAPK) kinase 3 (MKK3)-catalyzed kinase activity on p38^MAPK^. Subsequently, BI2B interrupted downstream pathway of p38^MAPK^-mitogen and stress-activated protein kinase-1 (MSK1)-CREB-MITF-M, and suppressed MITF-M-target melanogenic genes, encoding enzymes TYR, TYR-related protein-1 (TRP-1) and dopachrome tautomerase (DCT) in melanin biosynthesis, and encoding proteins PMEL17 and Rab27A in the transfer of pigmented melanosomes to the overlaying keratinocytes in the skin.

**Conclusion:** Targeting the MKK3-p38^MAPK^-MSK1-CREB-MITF-M pathway was suggested as a rationale to inhibit UV-B- or α-MSH-induced facultative melanogenesis and as a strategy to prevent acquired pigmentary disorders in the skin.

## Introduction

Imbalanced overproduction and patched distribution of heavily pigmented melanosomes can cause acquired pigmentary diseases, such as melasma and freckles 1. Facultative melanogenesis is inducible and determines the degree of hyperpigmentation in the skin, in which ultraviolet B (UV-B) irradiation and melanogenic hormones are major stimulating factors 2, 3. Natural product/chemical-based control of facultative melanogenesis has been applied for pharmacological treatment of acquired pigmentary diseases in the skin as well as for cosmetic purpose to obtain a lightener skin appearance 4.

UV-B irradiation from sunlight up-regulates the expression of melanogenic hormones, such as α-melanocyte-stimulating hormone (α-MSH), and stem cell factor at epidermal keratinocytes in the skin 2. In a paracrine fashion, melanogenic hormones from keratinocytes bind to their specific receptors in melanocytes, which transmit melanocyte-specific signals to express the microphthalmia-associated transcription factor M (MITF-M), thus regulating melanosome pigmentation and the transfer of pigmented melanosomes to keratinocytes at the overlaying epidermis to induce hyperpigmentation in the skin 2, 5. Moreover, UV-B irradiation onto melanocytes transmits melanogenic signals through mitogen-activated protein kinase (MAPK) family, such as p38^MAPK^, extracellular signal-regulated kinase (ERK) and c-Jun *N*-terminal kinase (JNK), in the expression of MITF-M 6, 7.

α-MSH binds to the extracellular domain of melanocortin 1 receptor (MC1R) at epidermal melanocytes in the skin, which initiates intracellular signals to increase cAMP levels via activation of adenylate cyclase 3. The second messenger cAMP binds to the regulatory subunit (R) of inactive protein kinase A (PKA) holoenzyme (R_2_C_2_) and dissociates the catalytic subunit (C), thus gaining the kinase activity of PKA 8. MITF-M is inducible in response to α-MSH stimulation or UV-B irradiation 5. The promoter region of MITF-M encodes several *cis*-acting consensus, including the cAMP response DNA element (CRE) and the lymphoid enhancer binding factor 1 (LEF1)-binding motifs 9-11. Transcriptional activation of MITF-M at the promoter level is maximally up-regulated by specific interactions between the CRE and the heteromer of CRE-binding protein (CREB) and CREB regulated transcription coactivators (CRTCs) as well as those between the LEF1-binding motifs and the heteromer of LEF1 and β-catenin 9-11. Nuclear CREB and LEF1 directly interact with corresponding *cis*-acting consensus in the promoter region of MITF-M 9-11. After translocation to the nucleus, CRTCs or β-catenin respectively coactivate CREB or LEF1 that already occupy the promoter region of MITF-M 9-11.

Transcriptional activities of CREB, CRTCs and β-catenin are tightly regulated by posttranslational modification of reversible phosphorylation and dephosphorylation 9, 10. PKA activity in the axis with cAMP directly phosphorylates nuclear CREB at the S133 residue, a co-substrate of the kinase activity of the mitogen- and stress-activated protein kinase-1 (MSK1) in the axis with p38^MAPK^ or that of p90 ribosomal S6 kinase (p90RSK) in the axis with ERK 12-14. The phosphor (p)-CREB is mostly dephosphorylated in the nucleus by protein phosphatase 2A, which cannot alter the affinity of p-CREB or CREB to the CRE in promoter region of MITF-M 15. In α-MSH-induced melanogenic programs, PKA directly phosphorylates the salt-inducible kinases (SIKs), the AMP-activated protein kinase (AMPK), and the glycogen synthase kinase-3β (GSK3β) 16-18. Kinase activities of SIKs and AMPK phosphorylate the conserved Ser residues of CRTCs (S64, S151 and S245 residues in CRTC1; S70, S171 and S275 residues in CRTC2; and S62, S162 and S273 residues in CRTC3) in the cytosol, which are reversibly dephosphorylated by calcineurin, a Ca^2+^/calmodulin-dependent protein phosphatase 19, 20. UV-B-induced, cAMP-dependent or anisomycin-activated kinase activity of JNK also phosphorylates the conserved Ser residues of CRTC3, leading to the tethering of p-CRTC3 with 14-3-3 protein and sequestering CRTC3 in the cytosol 21, 22. Kinase activity of ERK directly phosphorylates CRTC3 at the S391 residues, which facilitates the recruitment of calcineurin, and dephosphorylates p-CRTC3 at the S162 and S273 residues, thus resulting in the dissociation of 14-3-3 protein 20. In α-MSH-induced melanogenic programs, kinase activity of GSK3β in the axis with PKA phosphorylates β-catenin at the S675 residue in the cytosol, thus stabilizing p-β-catenin from degradation by ubiquitination and proteasome system 18, 23.

Human melanocytes are not simply melanin pigment-producing cells but also implicated in innate immunity through Toll-like receptors (TLRs) 24. Endotoxin lipopolysaccharide (LPS), an agonistic ligand of TLR4, stimulates nuclear translocation of nuclear factor-κB (NF-κB) after degradation of inhibitor of NF-κB (IκB) to up-regulate NF-κB-dependent expression of cytokines in immune and other cell types including melanocytes 24, 25. In melanocytes, LPS/TLR4 transmit intracellular signals to regulate MITF-M-dependent expression of melanogenic genes, in addition to NF-κB-associated innate immunity 24, 25.

**Figure 1:**
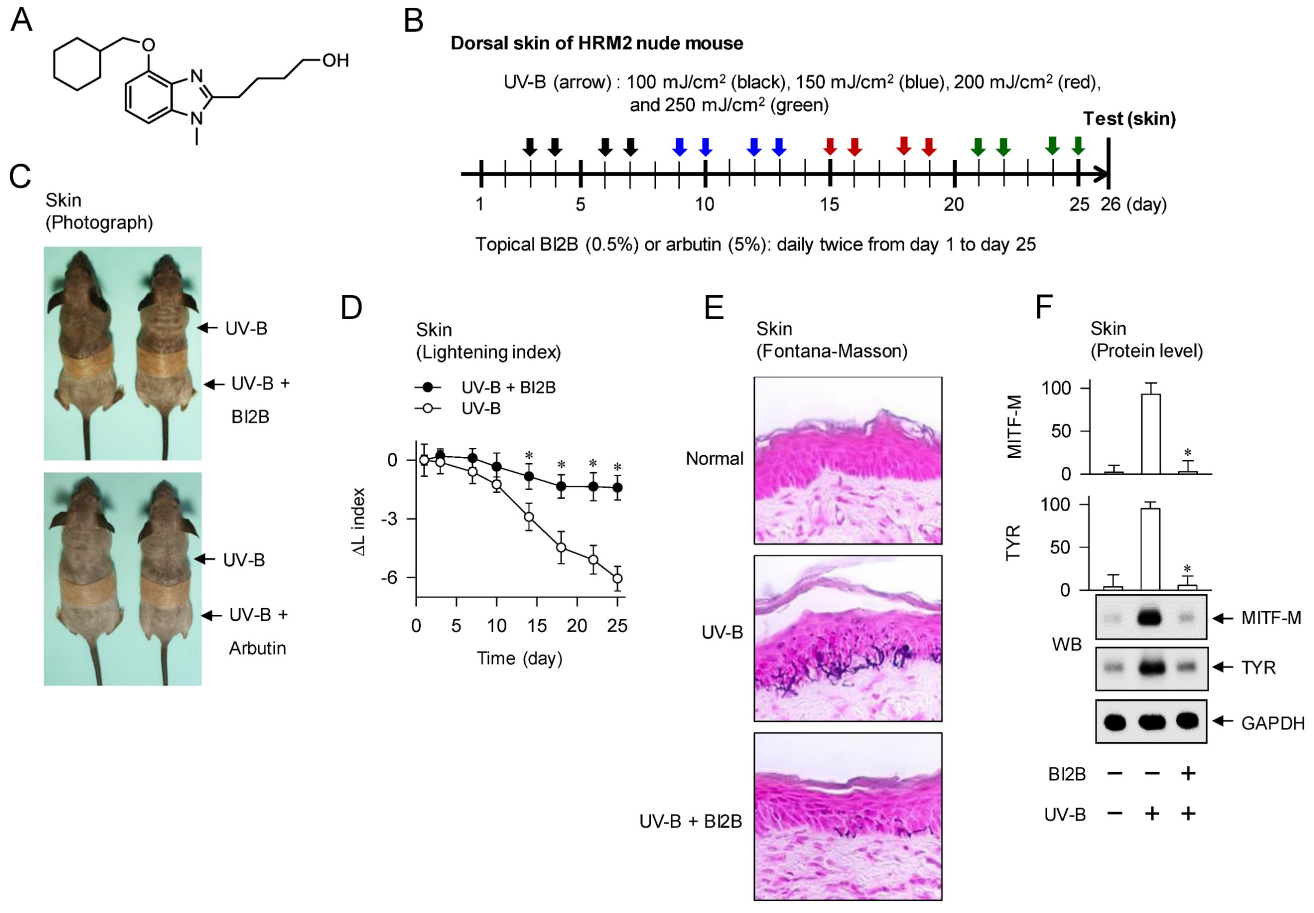

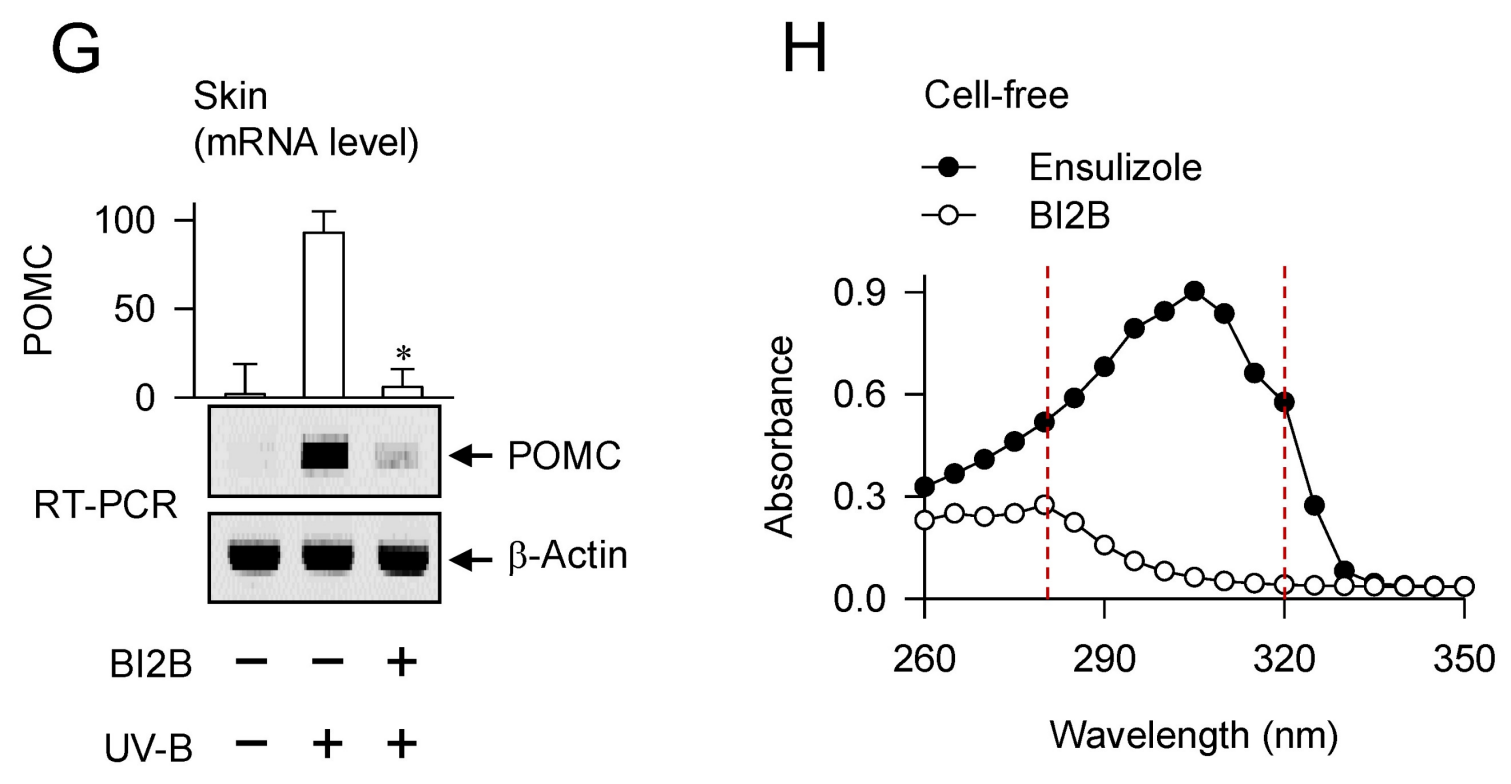
** Effect of BI2B on skin hyperpigmentation *in vivo*.** (A) Chemical structure of BI2B. (B) Experimental protocol of skin hyperpigmentation. The dorsal skins of HRM2 hairless mice were topically treated with BI2B (0.5%) or arbutin (5%) in a daily twice regimen for consecutive 25 days, and irradiated with increasing doses (100-250 mJ/cm^2^) of UV-B at the time points indicated by arrows. Melanogenic markers were examined in alive or biopsied skins at day 26. (C) BI2B inhibited UV-B irradiated visual hyperpigmentation in alive skins, as did arbutin. (D) BI2B restored UV-B-induced change of lightening (ΔL) index in alive skins during 25 days to normal status. (E) BI2B inhibited the deposit of melanin granules in UV-B-exposed and pigmented skin tissues. (F, G) BI2B suppressed the protein levels of MITF-M or TYR and mRNA levels of POMC in UV-B-exposed and pigmented skin tissues. (H) BI2B did not absorb UV-B light, in which ensulizole was employed as a positive control agent. Three independent tests with two mice per group. *P < 0.05 vs. UV-B alone.

In our previous work, a derivative of benzimidazole-2-butanol (BI2B, Figure 1A) inhibits LPS/TLR4-activated transcriptional activity of NF-κB in immune cells 26. Moreover, BI2B inhibited LPS/TLR4-induced melanin production in melanocytes (Figure S1). We then hypothesized that BI2B could negatively regulate intracellular signals of melanin production coupling with those of NF-κB activation. In the current study, we propose a molecular target of antimelanogenic activity by elucidating the inhibitory activity and mechanism of BI2B on facultative melanogenesis via UV-B irradiation or α-MSH stimulation. Topical treatment with BI2B ameliorated hyperpigmentation in UV-B-irradiated skins of mice. Moreover, BI2B inhibited melanin pigmentation in UV-B-irradiated co-cultures of keratinocyte and melanocyte cells and α-MSH-activated melanocytes. As a mechanism, BI2B primarily inhibited MAPK kinase 3 (MKK3)-catalyzed kinase activity on p38^MAPK^, thus interrupting the downstream pathway of p38^MAPK^-MSK1-CREB-MITF-M in α-MSH-induced melanogenic programs.

## Materials and Methods

### Drugs and chemicals

BI2B (purity, >95%) was prepared as described previously 26. Melanogenic stimuli were α-MSH (Sigma-Aldrich, M4125) as hormone; and *N*^6^,2'-*O*-dibutyryl-cAMP (db-cAMP; Sigma-Aldrich, D0627) as cAMP agonist. Pharmacological agents were arbutin (Sigma-Aldrich, A4256) as skin whitener; ensulizole (Sigma-Aldrich, 437166) as ingredient of sun blocker; KT 5720 (Sigma-Aldrich, K3761) as PKA inhibitor; SB2020190 (Sigma-Aldrich, S7067) as p38^MAPK^ inhibitor; SB203580 (Sigma-Aldrich, S8307) as p38^MAPK^ inhibitor; SB-747651A (Tocris, 4630) as MSK1 inhibitor; and RMM-46 (Tocris, 5909) as MSK1 inhibitor.

### Skin hyperpigmentation by UV-B irradiation

HRM-2 hairless mice were obtained from Central Lab Animals (Eumsung, Korea), and housed in the facility under relative humidity 55±5%, temperature 22±2°C, and a 12-h light-dark cycle. Experimental protocol of hyperpigmentation in the skin is shown in Figure 1B. BI2B (0.5%) or arbutin (5%) was dissolved in a vehicle of propylene glycol: ethyl alcohol: H_2_O (5: 3: 2) and topically treated to the dorsal skins of mice in a daily twice regimen for consecutive 25 days. Increasing doses (100-250 mJ/cm^2^) of UV-B were irradiated at the time points indicated by arrows. Lightening index of UV-B-exposed and pigmented skins in alive mice was measured once per every three days using a chromameter. The UV-B-exposed and pigmented skins were biopsied at day 26. Skin tissues were fixed in *p*-formaldehyde, embedded in paraffin, sectioned in a 5-μm thickness, and reacted with Fontana-Masson silver nitrate (Scytek, FMS-1) to stain melanin pigments in deposit. Protein extracts or total RNAs were separted from skin tissues, and subjected to Western blot (WB) or RT-PCR analysis. All procedures for animal study were approved as permission number CBNUR-1253-19 by the Animal Experimentation Ethics Committee of CBNU institute.

### Cell culture

B16F0 mouse melanoma cells (ATCC, CRL-6322) or HaCaT human keratinocyte cells (AddexBio, T0020001) were cultured in Dulbecco's modified Eagle's medium (DMEM; Sigma-Aldrich, D2902) containing 10% fetal bovine serum (Coring, 35-01-CV) and antibiotic-antimycotic solution (Gibco, 15240062) at 37°C in a 5% CO_2_ atmosphere. For co-culture, B16F0 cells were seeded on 6-well plates and HaCaT cells on the trans-well insert containing a pore size of 0.4 μm (Corning, 3412). HaCaT cells on the trans-well inserts were transferred to B16F0 cells in the 6-well plates, and cultured in the same condition as described above. Human epidermal melanocyte (HEM; Thermo Fischer Scientific, C1025C) was cultured in medium 254 (Gibco, M254) supplemented with melanocyte growth supplement (Gibco, S002) at 37°C in a 5% CO_2_ atmosphere.

### Western blot analysis

Protein extracts were prepared from skin tissues, HEM or B16F0 cells, resolved on sodium dodecyl sulfate (SDS)-acrylamide gels by electrophoresis, and transferred to the membranes of PVDF (Merck-Millipore, IPVH00010) using a semidry blotting apparatus. After blocking with 5% non-fat milk (BD Biosciences, 232100) in Tris-buffered saline (TBS) with Tween 20 (Biosesang, TR2007-100-74), blots were reacted with primary antibody overnight at 4°C. After washing with TBS containing Tween 20, blots were reacted with horseradish peroxidase (HRP)-labeled secondary antibody for 1-3 h and visualized the immune complex by chemiluminescence kit (GE Healthcare, RPN2232). Primary antibodies were anti-MITF-M (Abcam, ab12039); anti-TYR (Santa Cruz Biotechnology, sc-7833); anti-glyceraldehyde 3-phosphate dehydrogenase (GAPDH; Cell Signaling Technology, 5174); anti-p-CREB (Cell Signaling Technology, 9198); anti-CREB (Cell Signaling Technology, 9197); anti-p-CRTC1 (Cell Signaling Technology, 3359); anti-CRTC1 (Cell Signaling Technology, 2587); anti-p-β-catenin (Cell Signaling Technology, 9567); anti-β-catenin (Cell Signaling Technology, 9562); anti-SRY-box transcription factor 10 (SOX10; Santa Cruz Biotechnology, sc-365692); anti-lamin A (Santa Cruz Biotechnology, sc-20680); anti-p-p38^MAPK^ (Cell Signaling Technology, 9211); anti-p38^MAPK^ (Cell Signaling Technology, 9212); anti-p-ERK (Cell Signaling Technology, 9101); anti-ERK (Cell Signaling Technology, 9102); anti-p-JNK (Cell Signaling Technology, 9251); anti-JNK (Cell Signaling Technology, 9252); anti-p-MSK1 (Cell Signaling Technology, 9595); anti-MSK1 (Cell Signaling Technology, 3489); anti-p-MKK3/6 (Cell Signaling Technology, 9236); anti-MKK3/6 (Cell Signaling Technology, 8535); anti-tyrosinase (TYR)-related protein-1 (TRP-1; Santa Cruz Biotechnology, sc-10446); and anti-dopachrome tautomerase (DCT; Santa Cruz Biotechnology, sc-10451). Secondary antibodies were HRP-labeled anti-rabbit IgG (Thermo Fisher Scientific, 31460) and HRP-labeled anti-mouse IgG (Thermo Fisher Scientific, 31430).

### RT-PCR analysis

Total RNAs were extracted from skin tissues, HEM or B16F0 cells using NucleoZOL kit (Macherey-Nagel, 740404). Complementary DNA (cDNA) was prepared from 1 ug of total RNAs using a reverse transcription kit (Intron, 25087) and subjected to PCR analysis to determine the levels of each transcript, such as POMC, MITF-M, CREB, CRTC1, β-catenin, TYR, TRP-1, DCT, PMEL17, Rab27A or β-catenin. Nucleotide sequences of PCR primers were described in Table S1. RT-PCR condition was as follows: reverse transcription at 95°C for 3 min followed by 25-30 cycles of PCR at 94°C for 30 sec (denaturation), 50-60°C for 1 min (annealing), and 72°C for 1 min (extension). Amplified transcripts were resolved on agarose gels by electrophoresis and visualized by staining with EcoDye (Biofact, ES301).

### Quantification of melanin pigments

B16F0 or HEM cells were respectively seeded on 96-well or 6-well plates, and stimulated with 100 nM α-MSH or 3 mM db-cAMP for 72 h. Extracellular melanin pigments were collected from the medium. To extract intracellular melanin pigments, the cells were disrupted using 0.85 N NaOH and 20% dimethyl sulfoxide (DMSO; Sigma-Aldrich, 34869) at 80℃. The extracellular and intracellular melanin pigments were quantified by measuring the absorbance values at wavelength 405 nm.

### Cell viability assay

B16F0 or HEM cells were cultured with BI2B for 72 h, and reacted with 0.5 mg/ml 3-(4,5-dimethylthiazol-2-yl)-2,5-diphenyltetrazolium bromide (MTT; Sigma-Aldrich, M5655) for another 30 min. Insoluble formazan crystals were dissolved in 50% DMSO and their absorbance values were measured at wavelength 590 nm.

### Luciferase reporter assay

B16F0 cells were transfected with plasmid construct encoding firefly luciferase reporter fused to the promoter region of MITF-M (MITF-M-Luc) or that of TYR (TYR-Luc) along with *Renilla* control vector using lipofectamine kit (Invitrogen, 11668), and incubated for 48 h. The cells harboring each reporter plasmid were stimulated with 100 nM α-MSH in the presence of BI2B for 20 h, and harvested for dual-luciferase assays (Promega, E1910). The promoter activity of MITF-M or TYR was measured by Firefly luciferase activity, and normalized by *Renilla* luciferase activity, indicating the transfection efficiency.

#### Small interfering (si) RNA assay

The siRNAs against CREB, CRTC1 or β-catenin were supplied from Bioneer (Cheongju, Korea), and their nucleotide sequences were 5'-GAUUCACAGGAGUCUGUGG-3' (CREB), 5'-UGGACAGAGUAUAUCGUGA-3' (CRTC1), and 5'-CUGUUGGAUUGAUUCGAAA-3' (β-catenin). B16F0 cells were transfected with each siRNA using lipofectamine kit, and incubated for 48 h, and stimulated with α-MSH for 2 h. Total RNAs were subjected to RT-PCR analysis of MITF-M, CREB, CRTC1 and β-catenin as described above.

### Confocal microscopy

B16F0 cells were seeded on poly-Lys-coated glass slides, fixed with *p*-formaldehyde, and permeabilized with PBS containing 0.5% Triton X-100 (Biosesang, PR4007) for 10 min. The cells on glass slides were incubated with PBS containing 1% bovine serum albumin (BSA; Sigma-Aldrich, A9647), and reacted with each antibody against CREB, p-CREB or CRTC1. After washing, the cells on glass slides were stained with Alexa Fluor 488-conjugated secondary antibody (Invitrogen, A-11034) and also with Vecashield antifade medium containing 4',6-diamidino-2-phenylindole (DAPI; Vector Lab, H1200), and examined using confocal microscope (Carl Zeiss LSM 980).

### Kinase assay

To determine cellular PKA activity, lysates of B16F0 cells were reacted with kemptide (Promega, V5601) as PKA-specific peptide substrate, and 10 μCi [γ-^32^P]-ATP (Perkin Elmer, NEG002A) as a probe for 30 min at 30°C. To determine *in vitro* kinase activity of MKK3 in cell-free conditions, catalytically active rhMKK3 (SignalChem, M04-10G) was reacted with p38α^MAPK^ (SignalChem, M37-14G) as a substrate in presence of 10 μCi [γ-^32^P]-ATP as a probe for 30 min at 30°C. Similarly, catalytically active TGF-β-activated kinase 1 (rhTAK1)-TAK1 binding protein 1 (TAB1) (Promega, V4088) was reacted with major basic protein (MBP; SignalChem, M42-51) as an exogenic substrate in presence of 10 μCi [γ-^32^P]-ATP as a probe for 30 min at 30°C. The mixtures of kinase reactions were spotted on a P81 phosphocellulose paper and washed with 0.75% H_3_PO_4_ followed by 100% acetone. Radioactivity on the paper was determined as count per min (cpm) using scintillation counting to quantify the kinase activity.

### Statistical analysis

Results are expressed as the mean ± SEM of 3 independent experiments (n = 3). Data were statistically analyzed using the analysis of variance (ANOVA) or Student's *t*-test. *P* < 0.05 were considered as statistically significant.

## Results

### Amelioration of UV-B-irradiated hyperpigmentation in the skin of mice by BI2B

To examine antimelanogenic activity of BI2B *in vivo*, we employed hyperpigmentation in UV-B-irradiated skins of mice. The dorsal skins of HRM-2 hairless mice were topically treated with BI2B (0.5%) or arbutin (5%, a dose approved by Korea FDA) in a daily twice regimen for consecutive 25 days, and stimulated with UV-B irradiation at the time points indicated by arrows (Figure 1B). UV-B-exposed and pigmented skins were biopsied at day 26, and their melanogenic markers were evaluated.

Topical treatment with BI2B decreased visual hyperpigmentation in UV-B-irradiated alive skins at day 26, as did that with arbutin (Figure 1C). Arbutin is a Korea FDA-approved lightening drug, thus employing it as a positive control agent in this study. The percentage change of lightening (ΔL) index, inversely correlating with the degree of hyperpigmentation, was gradually decreased in UV-B-irradiated alive skins during 25 days, which was restored to basal status by treatment with BI2B (Figure 1D). UV-B-irradiated skins were then biopsied at day 26. To examine the deposit of melanin granules, skin tissues were embedded in paraffin, sectioned in a 5-μm thickness, and stained with Fontana-Masson silver nitrate. Treatment with BI2B decreased the content of black-colored melanin granules in UV-B-irradiated skins, more evident in the border between epidermis and dermis where melanocytes produce pigmented melanosomes (Figure 1E). The protein or mRNA levels of melanogenic markers, such as MITF-M, TYR and proopiomelanocortin (POMC) were up-regulated in UV-B-irradiated skins, which were suppressed by treatment with BI2B (Figure 1F and G). However, BI2B did not absorb the UV-B light at wavelengths 280-320 nm, in which ensulizole is a UV-B-protecting ingredient and was employed as a positive control agent (Figure 1H). These results suggest that topical treatment with BI2B could ameliorate hyperpigmentation in UV-B-irradiated skins of mice.

### Inhibition of UV-irradiated, α-MSH-induced, or cAMP-dependent melanin production in melanocyte culture by BI2B

α-MSH is secreted from keratinocytes and melanocytes at the epidermis of skin after posttranslational processing of the precursor protein POMC that is up-regulated in response to UV-B 2, 27. In paracrine and autocrine fashions, α-MSH binds to its receptor MC1R at melanocytes, which stimulates cAMP-dependent melanin production to pigment the melanosomes 3, 8.

**Figure 2:**
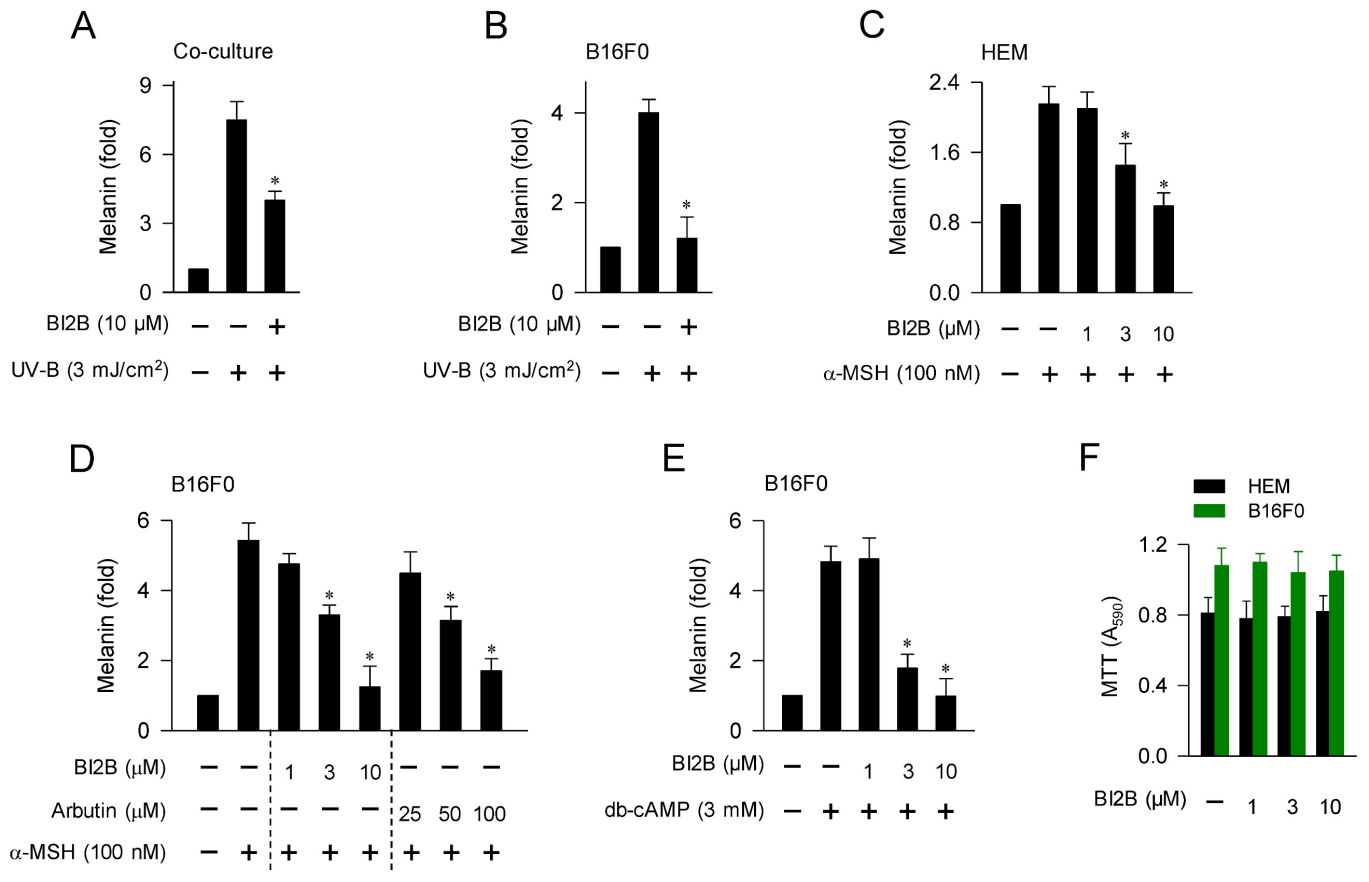
** Effect of BI2B on melanin production in cell-culture model.** (A) Co-cultures of HaCaT keratinocyte and B16F0 melanoma cells were stimulated with a single irradiation of UV-B and treated with BI2B for 72 h. BI2B inhibited melanin production in UV-B-irradiated co-cultures. (B) Monocultures of B16F0 cells were stimulated with a single irradiation of UV-B and treated with BI2B for 72 h. BI2B inhibited melanin production in UV-B-irradiated monocultures. (C-E) HEM or B16F0 cells were stimulated with α-MSH or db-cAMP in the presence of BI2B for 72 h. BI2B inhibited α-MSH- or db-cAMP-induced melanin production. (F) HEM or B16F0 cells were incubated with BI2B for 72 h. BI2B did not alter MTT assay-based cell viability. *P < 0.05 vs. UV-B alone, α-MSH alone or db-cAMP alone.

We then examined whether antimelanogenic activity of BI2B *in vivo* could be translated in the facultative melanogenesis *in vitro* using cell-culture models. Upon a single irradiation with UV-B, co-cultures of HaCaT keratinocyte and B16F0 melanoma cells stimulated melanin production, which was inhibited by treatment with BI2B (Figure 2A). In this co-culture model, melanogenic hormones including α-MSH might be secreted from HaCaT cells and interacted with their receptors including MC1R in B16F0 cells to stimulate melanin production. Monocultures of HEM or B16F0 cells also stimulated melanin production upon a single irradiation with UV-A or UV-B, which were also inhibited by treatment with BI2B (Figure 2B; Figure S2). In this monoculture model, UV-B might stimulate α-MSH-induced melanin production in an autocrine manner or might activate MAPKs-signaled melanogenic programs in melanocytes. Treatment with BI2B inhibited α-MSH-induced melanin production in HEM or B16F0 cells (Figure 2C and D), suggesting its antimelanogenic activity regardless of the melanocyte origins from human and mouse. BI2B showed more effective activity (approximately 10-fold) than arbutin in the comparison with their IC_50_ values on α-MSH-induced melanin production (Figure 2D), which was applied to select the dose of BI2B *in vivo* (Figure 1B). Treatment with BI2B also inhibited db-cAMP-induced melanin production in B16F0 cells (Figure 2E). The db-cAMP was employed as a cAMP agonist. However, BI2B at 1-10 μM did not alter MTT assay-based viability of HEM or B16F0 cells (Figure 2F), excluding from non-specific cytotoxicity. These results suggest that BI2B could inhibit various models of facultative melanogenesis in melanocyte cultures. We then focused on α-MSH-induced melanogenic programs in melanocytes to elucidate the mechanism of BI2B on antimelanogenic activity.

### Down-regulation of MITF-M expression at the promoter level in α-MSH-induced melanogenic programs by BI2B

**Figure 3:**
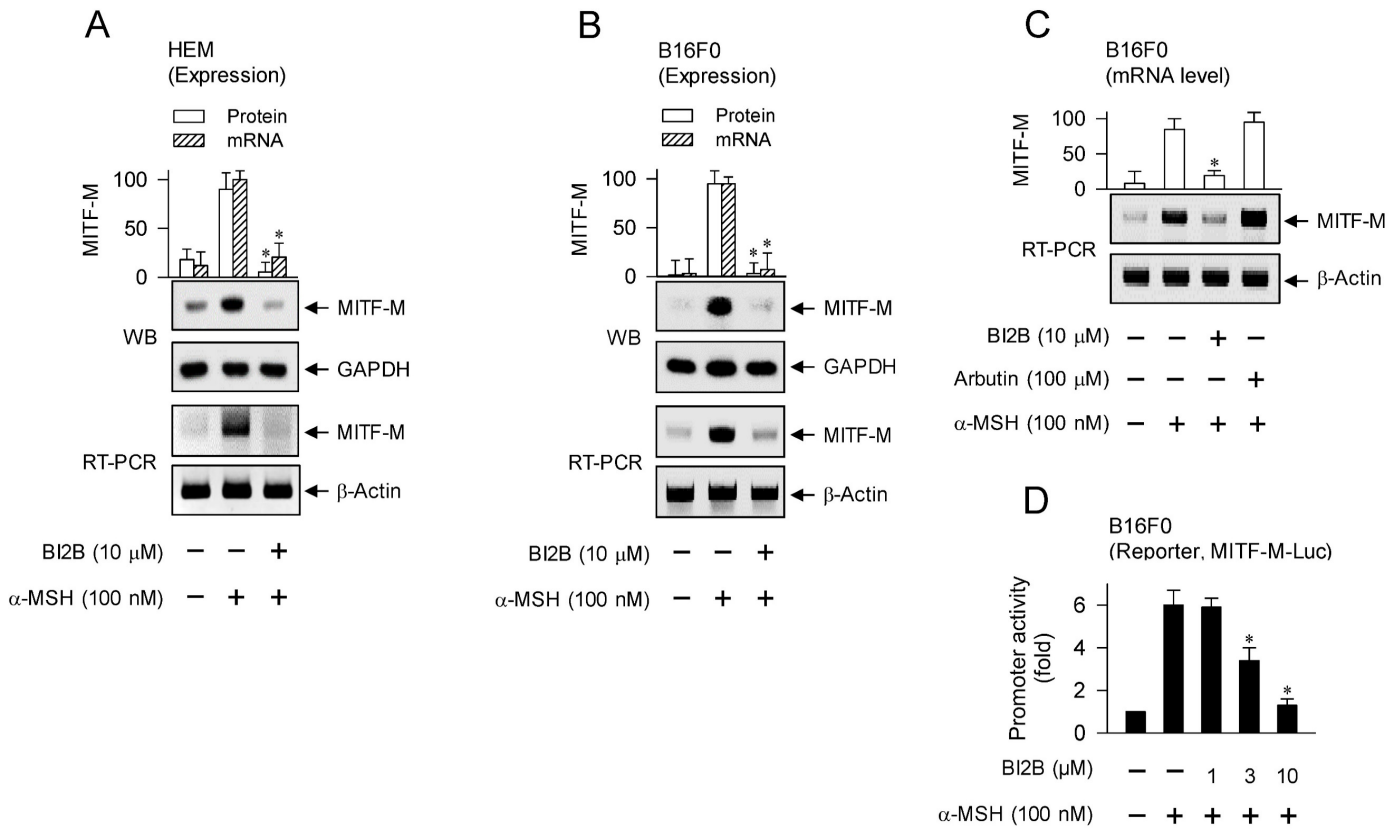
** Effect of BI2B on α-MSH-induced MITF-M expression.** HEM or B16F0 cells were pretreated with BI2B for 2 h and stimulated with α-MSH for another 2 h (mRNA levels at A-C) or 4 h (protein levels at A, B) in the presence of BI2B. (A, B) BI2B suppressed α-MSH-induced expression of MITF-M in melanocytes. (C) Arbutin did not alter α-MSH-induced transcription of MITF-M. (D) B16F0 cells were transfected with MITF-M-Luc reporter, and stimulated with α-MSH in the presence of BI2B for 20 h. BI2B inhibited α-MSH-induced promoter activity of MITF-M. *P < 0.05 vs. α-MSH alone.

MITF-M is inducible in melanocytes and functions as a master transcription factor that regulates the biogenesis, pigmentation and transfer of melanosomes 5, 10, 28. We evaluated whether BI2B could affect the expression of MITF-M in α-MSH-activated melanocyte cultures, since topical treatment with BI2B suppressed the protein levels of MITF-M in UV-B-irradiated and pigmented skins of mice (Figure 1F, upper). HEM or B16F0 cells increased the protein and mRNA levels of MITF-M in response to α-MSH, which were suppressed by treatment with BI2B (Figure 3A and B). In contrast, treatment with arbutin did not alter the mRNA levels of MITF-M in α-MSH-activated B16F0 cells (Figure 3C), suggesting that BI2B may be different in the antimelanogenic mechanism from arbutin.

Transcriptional regulation of MITF-M by BI2B was further characterized using luciferase reporter assay-based promoter activity. B16F0 cells were transfected with MITF-M-Luc, a plasmid containing the promoter region (-2200/+95) of MITF-M and the reporter of firefly luciferase. B16F0 cells harboring MITF-M-Luc increased firefly luciferase activity in response to α-MSH, reporting the promoter activity of MITF-M, which was inhibited by treatment with BI2B (Figure 3D). These results suggest that BI2B could regulate the expression of MITF-M at the promoter level.

**Figure 4:**
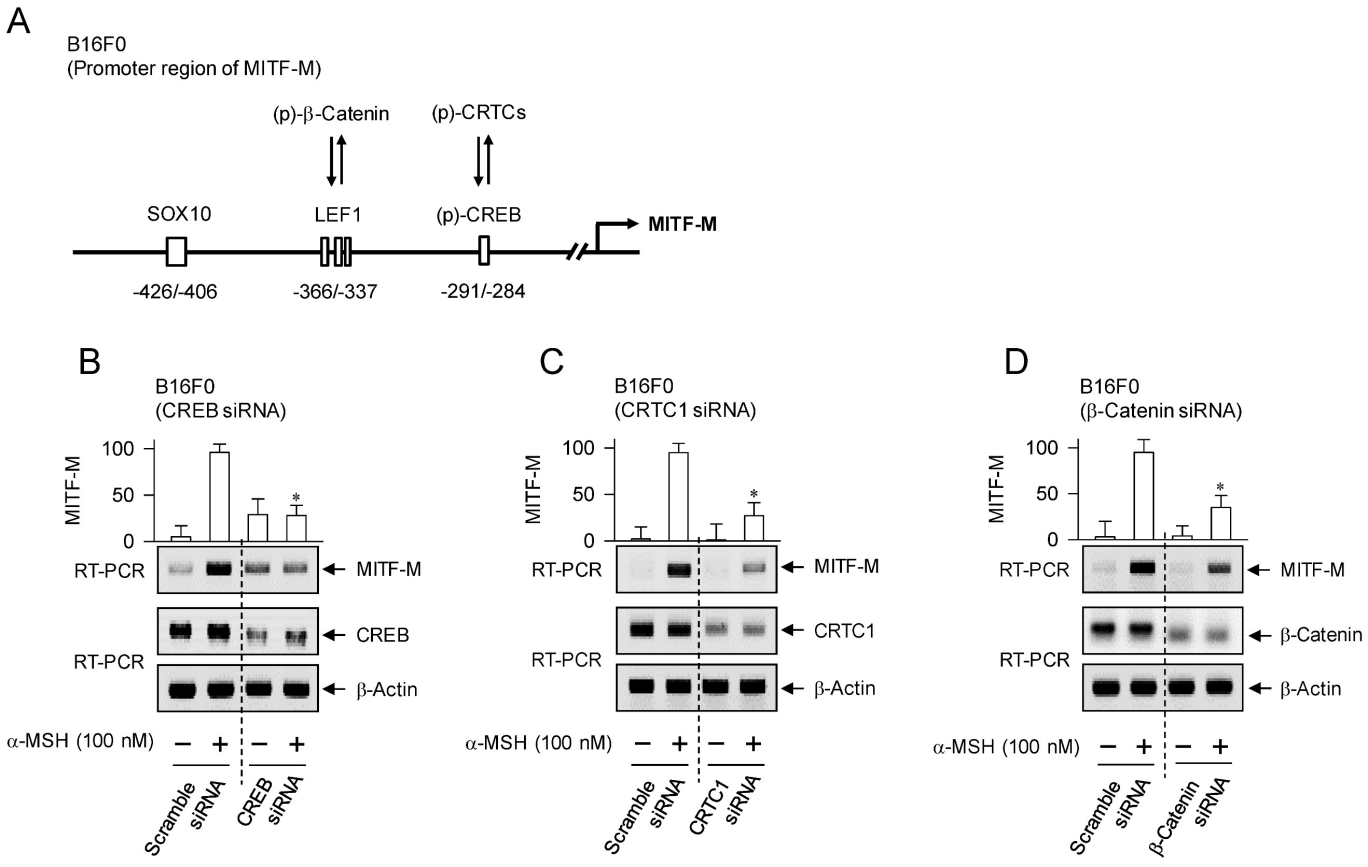
** Transcriptional activators on MITF-M promoter in response to α-MSH.** (A) An illustrated structure of MITF-M promoter: transcriptional activators and their *cis*-acting DNA elements. CREB and LEF1 constitutively interact with the corresponding *cis*-acting DNA elements. After nuclear translocation, CRTCs and β-catenin coactivate CREB and LEF1, respectively. Transcriptional activities of CREB, CRTCs and catenin are regulated by posttranslational modification of reversible phosphorylation and dephosphorylation. (B-D) B16F0 cells were transfected with each siRNA, incubated for 48 h and stimulated with α-MSH for 2 h. Knockdown of CREB, CRTC1 or β-catenin suppressed α-MSH-induced transcription of MITF-M. *P < 0.05 vs. scramble siRNA plus α-MSH.

The promoter region of mouse MITF-M encodes several *cis*-acting elements, such as the CRE at -291/-284, LEF1-binding sites at -366/-337 and SOX10-binding site at -426/-406 11, as represented in Figure 4A. After translocation from the cytosol to the nucleus, CRTCs and β-catenin coactivate CREB and LEF1 that already occupy the corresponding *cis*-acting elements on MITF-M promoter 9, 10. Each CRTC in the nucleus can dimerize with CREB, which makes more tight occupancy of CREB on the promoter 29. Moreover, another domain of CRTC interacts with basal transcription factor TFIID in the transcription machinery, thus facilitating the recruitment of RNA polymerase II onto the promoter 10, 29. Nuclear β-catenin dimerizes with LEF1 that is usually surrounded by transcriptional corepressors, which induces conformational changes leading to the dissociation of corepressor complex from LEF1, and alters a heteromer of LEF1/β-catenin to become transcriptional activator of MITF-M promoter 10, 18, 23. Melanocyte-specific SOX10 cooperates with ubiquitous CREB/CRTCs, which restricts the expression of MITF-M in melanocytes 30. To examine which transcription factors could be associated with the expression of MITF-M in response to α-MSH, gene-specific siRNA assay was employed. As shown in Figure 4B-D, knockdown of CREB, CRTC1 or β-catenin suppressed the transcription of MITF-M in α-MSH-activated B16F0 cells. These results suggest that CREB/CRTCs and LEF1/β-catenin could be responding to α-MSH-induced melanogenic programs as transcriptional activators of MITF-M promoter.

### Inhibition of CREB phosphorylation but not nuclear translocation of CREB, CRTC1, β-catenin or SOX10 in α-MSH-induced expression of MITF-M by BI2B

Melanogenic programs in response to α-MSH increase the phosphorylation of CREB coupling with the dephosphorylation of CRTCs for transcriptional activation of MITF-M through the complex of CREB/CRTCs 9, 10. Phosphorylation of CREB at the S133 residue promotes its affinity with CREB-binding protein (CBP) and paralogue p300 (CBP/p300), thus recruiting CBP/p300 onto the promoter region of MITF-M, where CBP/p300 catalyze the acetylation of nucleosomal histones for disassembly, thus becoming as transcriptionally active in chromatin structure 31, 32. Phosphorylation of CRTCs, including p-CRTC1 at the S151 residue, sequesters them in the cytosol, but dephosphorylation of them is essentially required for nuclear translocation 16, 33. Phosphorylation of β-catenin at the S675 residue stabilizes its conformation from proteasome-mediated degradation, resulting in its accumulation in the cytosol 18, 23. The p-β-catenin is then allowed to translocate into the nucleus, and interacts with LEF1 for transcriptional activation of MITF-M promoter 10, 18, 23.

**Figure 5:**
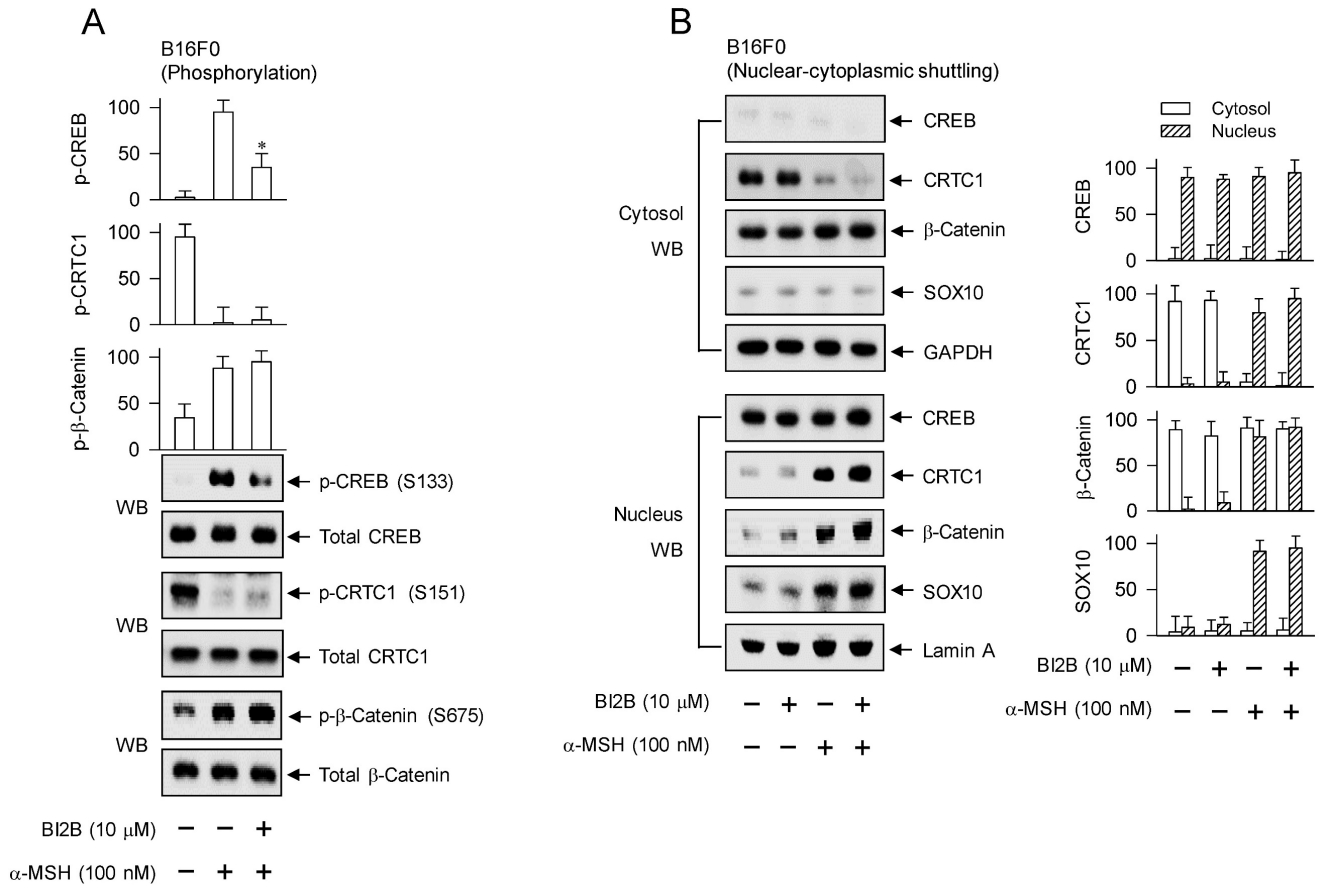
** Effects of BI2B on the phosphorylation of CREB and the nuclear-cytosolic shuttling of CREB, CRTC1, β-catenin or SOX10 in response to α-MSH.** B16F0 cells were pretreated with BI2B for 2 h and stimulated with α-MSH for 30 min (A) or 1 h (B) in the presence of BI2B. (A) BI2B inhibited α-MSH-induced phosphorylation of CREB but not those of CRTC1 and β-catenin. (B) BI2B did not alter α-MSH-induced nuclear translocation of CREB, CRTC1, β-catenin or SOX10. *P < 0.05 vs. α-MSH alone.

After stimulation with α-MSH, B16F0 cells increased the phosphorylation of CREB at the S133 residue, decreased that of CRTC1 at the S151 residue, and increased that of β-catenin at the S675 residue (Figure 5A). Treatment with BI2B decreased the levels of p-CREB but did not alter those of p-CRTC1 and p-β-catenin in α-MSH-activated B16F0 cells (Figure 5A). HEM cells also increased the phosphorylation of CREB coupling with dephosphorylation of CRTC1 in response to α-MSH (Figure S3), suggesting that phosphorylation circuits on CREB and CRTCs may be commonly operated in human and mouse melanocytes. Treatment with BI2B consistently inhibited the phosphorylation of CREB but bypassed the dephosphorylation of CTRC1 in α-MSH-activated HEM cells (Figure S3). Moreover, B16F0 cells increased the protein and mRNA levels of SOX10 in response to α-MSH, which were unaffected by treatment with BI2B (Figure S4A and B).

To examine nuclear translocation of the transcription factors in response to α-MSH, cytosolic and nuclear fractions were separated and then subjected to Western blot analysis with anti-CREB, anti-CRTC1, anti-β-catenin or anti-SOX10 antibody (Figure 5B). Anti-CREB antibody could react with dephosphorylated and phosphorylated CREBs, anti-CRTC1 antibody with dephosphorylated and phosphorylated CRTC1s, or anti-β-catenin antibody with dephosphorylated and phosphorylated β-catenins. GAPDH was employed as a marker of the cytosol and lamin A as that of the nucleus. Both dephosphorylated CREB in the presence of medium alone and phosphorylated CREB under stimulation with α-MSH were localized in the nucleus (Figure 5B). Phosphorylated CRTC1 in the presence of medium alone was found in the cytosol but dephosphorylated CRTC1 under stimulation with α-MSH was translocated to the nucleus (Figure 5B). Dephosphorylated β-catenin in the presence of medium alone was present in the cytosol but phosphorylated β-catenin under stimulation with α-MSH was partitioned between the nucleus and the cytosol (Figure 5B). SOX10 was localized in the nucleus after up-regulating its expression in response to α-MSH (Figure 5B; Figure S4A and B). Treatment with BI2B did not alter nuclear-cytosolic shuttling of CREB, CRTC1, β-catenin or SOX10 in α-MSH-activated B16F0 cells (Figure 5B).

**Figure 6:**
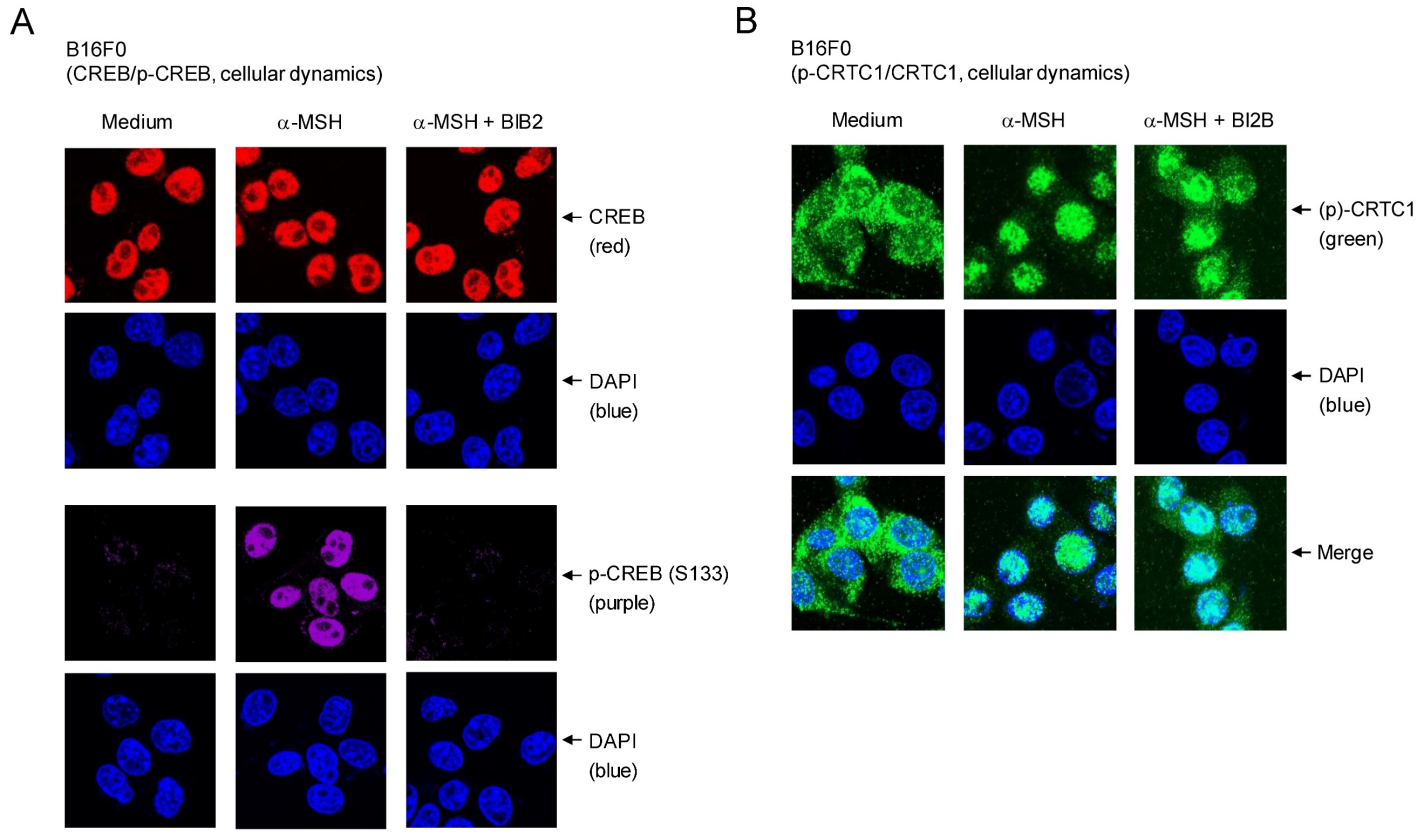
** Effects of BI2B on cellular dynamics of CREB and CRTC1 in response to α-MSH.** B16F0 cells were pretreated with BI2B for 2 h and stimulated with α-MSH for 1 h in the presence of BI2B. (A) Both CREB (red) in the presence of medium and p-CREB (purple) under stimulation with α-MSH were overlapped with the location of DAPI (blue), staining nucleic acids in the nucleus. BI2B inhibited α-MSH-induced phosphorylation of CREB but did not alter localization of CREB or p-CREB in the nucleus. (B) p-CRTC1 (green) in the presence of medium alone was stained with anti-CRTC1 antibody and predominantly localized in the cytosol. Dephosphorylated CRTC1 (green) under stimulation with α-MSH was found in the nucleus. BI2B did not alter α-MSH-induced nuclear translocation of CRTC1.

Cellular dynamics of CREB and CRTC1 in response to α-MSH were further characterized using confocal microscopy. Location of dephosphorylated CREB (red) was overlapped with that of DAPI (blue), staining nucleic acids in the nucleus (Figure 6A). B16F0 cells increased nuclear levels of p-CREB (purple) after stimulation with α-MSH (Figure 6A). Treatment with BI2B inhibited the phosphorylation of CREB in the nucleus of α-MSH-activated B16F0 cells but did not alter the localization of CREB or p-CREB in the nucleus (Figure 6A). On the other hand, p-CRTC1 (green) was stained with anti-CRTC1 antibody and mainly found in the cytosol of B16F0 cells in the presence of medium alone, but dephosphorylated CRTC1 (green) under stimulation with α-MSH was translocated to the nucleus (Figure 6B), suggesting that nuclear-cytosolic shuttling of CRTC1 and p-CRTC1 could be regulated by their reversible phosphorylation and dephosphorylation. Treatment with BI2B did not alter nuclear entry of dephosphorylated CRTC1 in α-MSH-activated B16F0 cells (Figure 6B). Overall, BI2B could inhibit the phosphorylation of CREB in α-MSH-induced melanogenic programs through the expression of MITF-M. However, BI2B did not alter the dephosphorylation of CRTC1, the phosphorylation of β-catenin, the up-regulation of SOX10, and the nuclear-cytosolic shuttling of CREB, CRTC1, β-catenin or SOX10 in α-MSH-activated melanocyte cultures.

### Interruption of p38^MAPK^-MSK1 axis but not cAMP-PKA axis in α-MSH-induced melanogenic programs by BI2B

**Figure 7:**
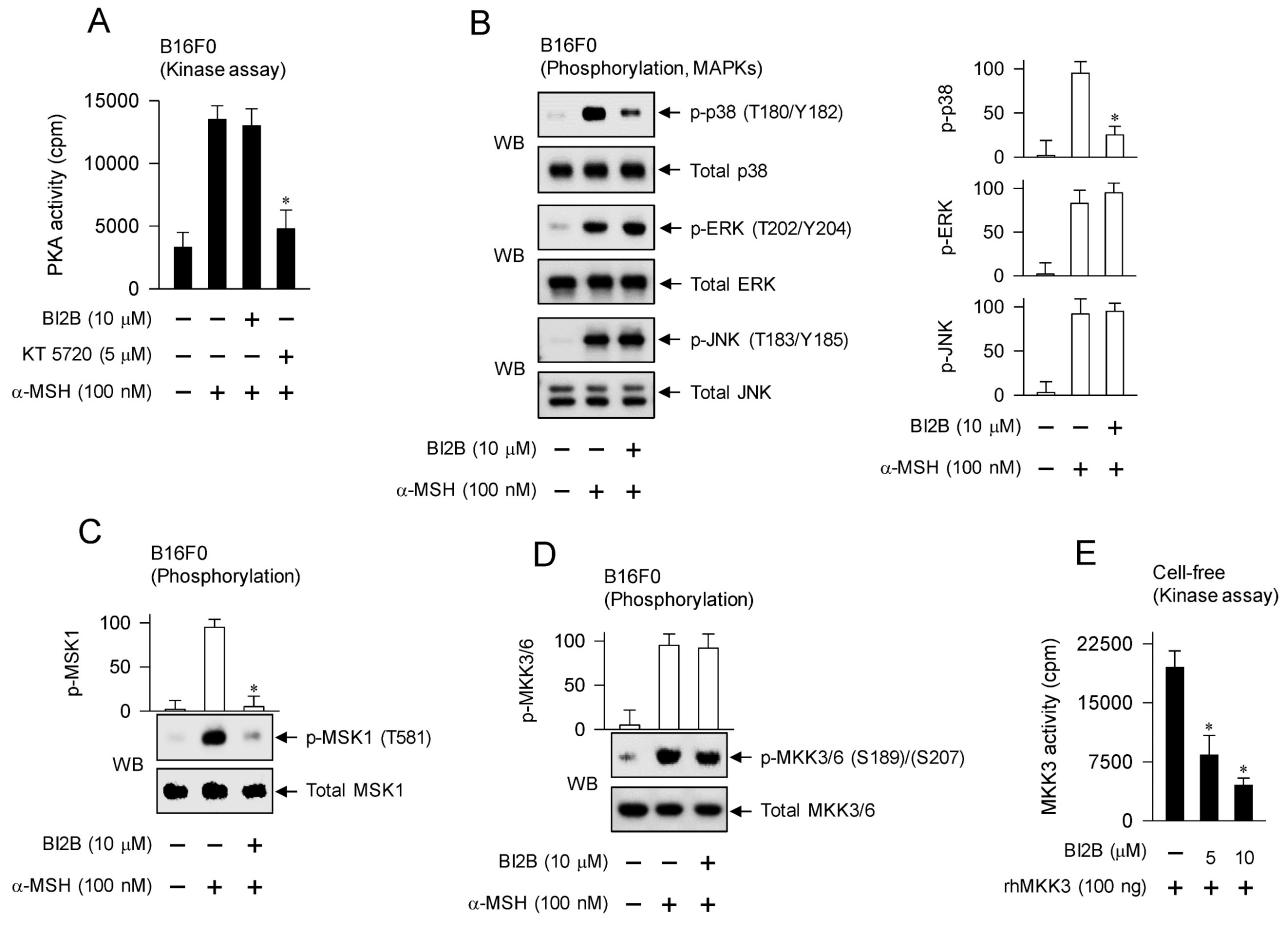
** Effects of BI2B on cAMP-PKA and p38^MAPK^-MSK1 axes in α-MSH-induced melanogenic programs.** B16F0 cells were pretreated with BI2B for 2 h and stimulated with α-MSH for 30 min in the presence of BI2B. (A) BI2B did not alter α-MSH-induced PKA activity. (B) BI2B inhibited α-MSH-induced phosphorylation of p38^MAPK^ but did not alter those of ERK and JNK. (C) BI2B inhibited α-MSH-induced phosphorylation of MSK1. (D) BI2B did not alter α-MSH-induced phosphorylation of MKK3/6. (E) Catalytically active rhMKK3 was treated with BI2B for 10 min, and its kinase activity was measured in cell-free reactions. BI2B inhibited MKK3-catalyzed kinase activity on p38^MAPK^. *P < 0.05 vs. α-MSH alone or rhMKK3 alone.

α-MSH binds to its receptor MC1R on the surface of melanocytes, which increases intracellular levels of cAMP, and activates PKA as well as MAPKs, thus up-regulating the expression of MITF-M in the melanogenic programs 3. B16F0 cells increased the kinase activity of PKA after stimulation with α-MSH, which was unaffected by treatment with BI2B but inhibited by that with KT 5720 (Figure 7A). KT 5720 was employed as an inhibitor of PKA-catalyzed kinase activity. From these results, BI2B might bypass cAMP-PKA axis-mediated signals to the expression of MITF-M, such as PKA-catalyzed phosphorylation (activation) of CREB 12, PKA-SIKs axis- or PKA-AMPK axis-mediated phosphorylation (inactivation) of CRTCs 16, 17, and PKA-GSK3β axis-mediated phosphorylation (activation) of β-catenin 18, 23.

We then evaluated whether BI2B could affect MAPKs-mediated signals to the expression of MITF-M in the melanogenic programs. B16F0 cells increased the phosphorylation of p38^MAPK^ at the T180 and Y182 residues, that of ERK at the T202 and Y204 residues, and that of JNK at the T183 and Y185 residues after stimulation with α-MSH (Figure 7B). Treatment with BI2B decreased the levels of p-p38^MAPK^ but did not alter those of p-ERK and p-JNK in α-MSH-activated B16F0 cells (Figure 7B). From these results, BI2B might bypass ERK- and JNK-dependent expression of MITF-M, such as ERK-MSK1 axis- or ERK-p90RSK axis-mediated phosphorylation (activation) of CREB 14, 34, ERK-calcineurin axis-mediated dephosphorylation (activation) of CRTCs 20, and JNK-catalyzed phosphorylation (inactivation) of CRTCs 21.

BI2B had inhibited not only the activation of CREB through phosphorylation (Figure 5A; Figure 6A) but also the activation of p38^MAPK^ in α-MSH-activated melanocyte cultures (Figure 7B). We then considered MSK1 as a candidate of linker between p38^MAPK^ and CREB, since MSK1 can be activated by p38^MAPK^-catalyzed phosphorylation 35, 36, and kinase activity of MSK1 phosphorylates CREB 13, 37. B16F0 cells increased the phosphorylation of MSK1 at the T581 residue after stimulation with α-MSH, which was inhibited by treatment with BI2B (Figure 7C).

To elucidate a primary target by BI2B, we examined the upstream kinases that activate p38^MAPK^. MKK3/6 can directly phosphorylate p38^MAPK^ 35, 36. B16F0 cells increased the phosphorylation of MKK3 at the S189 residue and that of MKK6 at the S207 residue after stimulation with α-MSH (Figure 7D). Treatment with BI2B did not alter α-MSH-induced phosphorylation (activation) of MKK3/6 at all (Figure 7D). To elucidate whether BI2B could directly affect catalytic activity of MKK3, we employed *in vitro* kinase assays. Treatment with BI2B inhibited rhMKK3-catalyzed phosphorylation of GST-p38α^MAPK^ in cell-free conditions (Figure 7E). In another, kinase activity of TAK1, a MAPK kinase kinase (MAPKKK), can phosphorylate MKK3/6 and lead to the activation of p38^MAPK^ activity 35. Treatment with BI2B did not alter catalytically active rhTAK1-TAB1-catalyzed kinase activity in cell-free reactions (Figure S5). These results suggest that BI2B could primarily inhibit the MKK3-catalyzed kinase activity on p38^MAPK^, thus interrupting the downstream pathway of p38^MAPK^-MSK1-CREB-MITF-M in α-MSH-induced melanogenic programs.

### Interruption of α-MSH-induced melanogenic programs by specific inhibitors of p38^MAPK^ and MSK1

**Figure 8:**
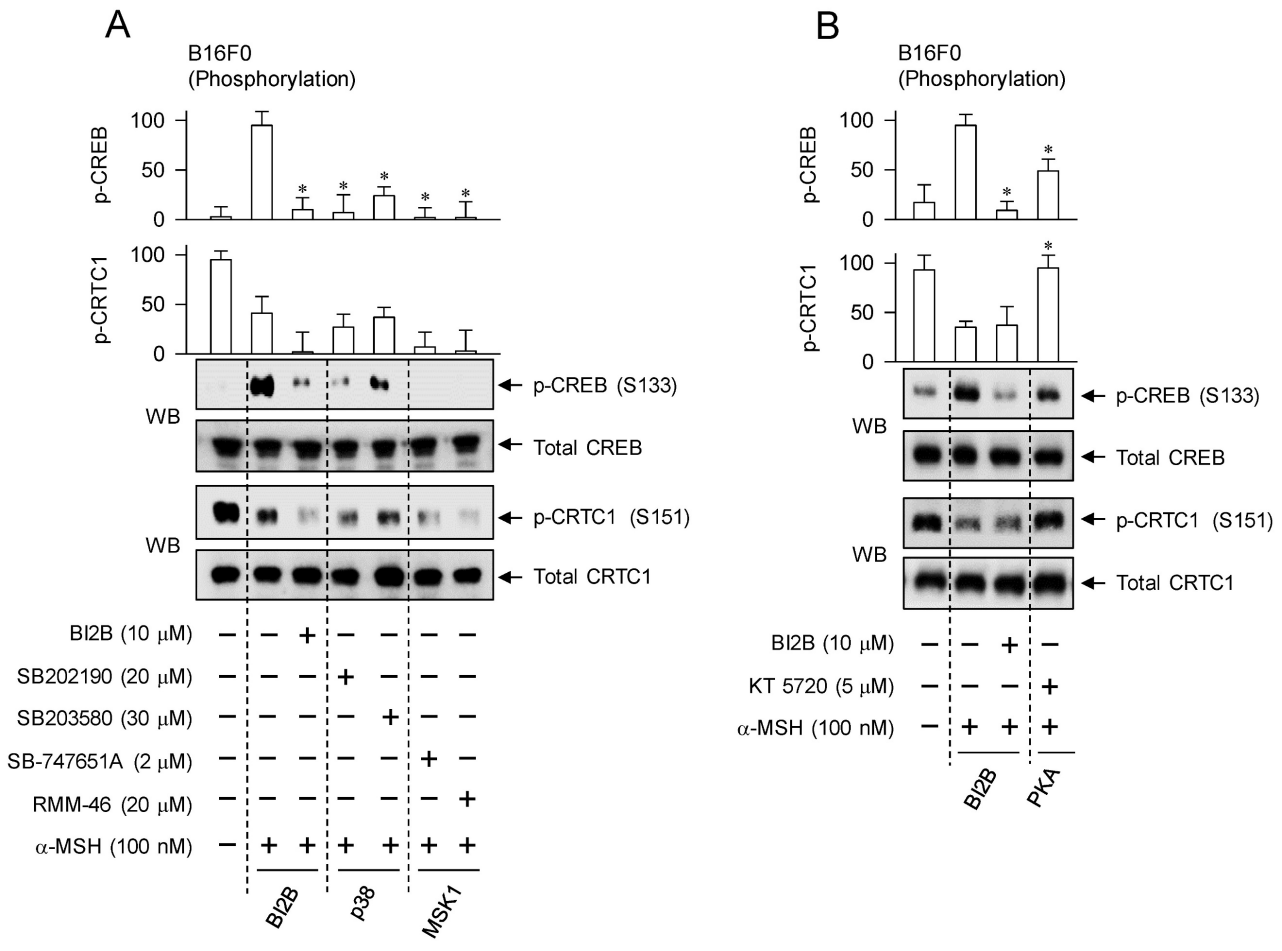

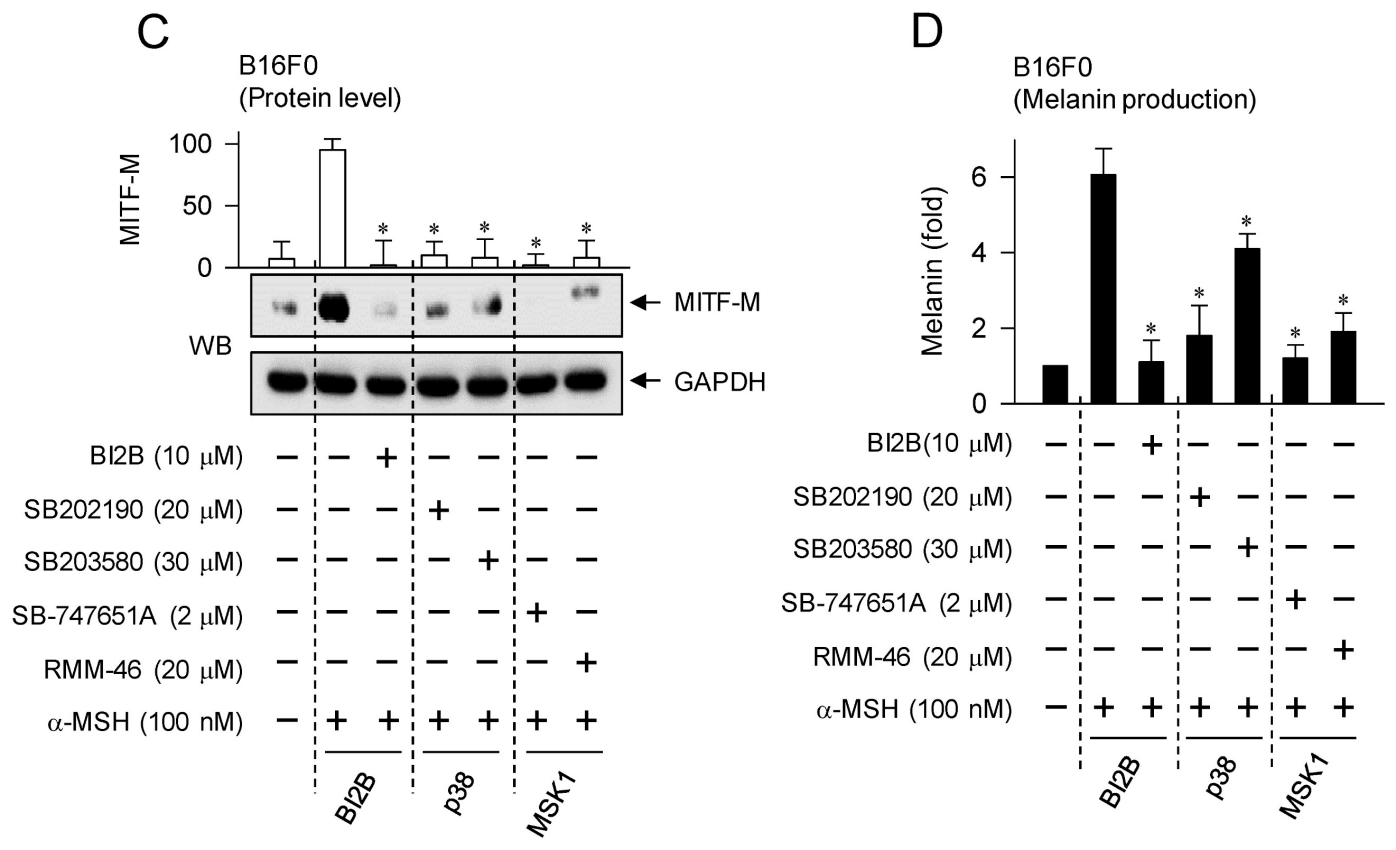
** Effects of p38^MAPK^ and MSK1 inhibitors on α-MSH-induced melanogenic programs.** SB202190 and SB203580 were employed as p38^MAPK^ inhibitors, SB-747651A and RMM-46 as MSK1 inhibitors, and KT 5720 as a PKA inhibitor. B16F0 cells were pretreated with each kinase inhibitor for 2 h and stimulated with α-MSH for 30 min (A, B), 4 h (C) or 72 h (D) in the presence of each kinase inhibitor. (A) Both p38^MAPK^ and MSK1 inhibitors decreased the phosphorylation of CREB in response to α-MSH but bypassed the dephosphorylation of CRTC1, as did BI2B. (B) PKA inhibitor decreased α-MSH-induced phosphorylation of CREB coupling with dephosphorylation of CRTC1. (C) Both p38^MAPK^ and MSK1 inhibitors suppressed α-MSH-induced protein levels of MITF-M, as did BI2B. (D) Both p38^MAPK^ and MSK1 inhibitors decreased α-MSH-induced melanin production, as did BI2B. *P < 0.05 vs. α-MSH alone.

We confirmed the importance of α-MSH-induced p38^MAPK^-MSK1-CREB-MITF-M pathway in the melanogenic programs using chemical inhibitors of p38^MAPK^ and MSK1. Treatment with small-molecule inhibitors of p38^MAPK^ or MSK1 decreased the phosphorylation of CREB in α-MSH-activated B16F0 cells but bypassed the dephosphorylation of CRTC1, as did that with BI2B (Figure 8A). Pyridinylimidazole derivatives SB202190 and SB203580 were employed as ATP-competitive inhibitors of p38^MAPK^-catalyzed kinase activity 38. Structurally unrelated chemicals SB-747651A and RMM-46 were employed as MSK1 inhibitors, in which SB-747651A targets to the kinase activity of *N*-terminal kinase domain on protein substrates including CREB, and RMM-46 to *C*-terminal kinase domain-catalyzed autophosphorylation (activation) of *N*-terminal kinase domain 37.

However, treatment with PKA inhibitor (KT 5720) partially inhibited the phosphorylation of CREB but completely restored the dephosphorylation of CRTC1 to basal status in α-MSH-activated B16F0 cells (Figure 8B). KT 5720 suppresses α-MSH-induced expression of MITF-M in melanogenic programs 39, and inhibited melanin production in α-MSH- or db-cAMP-activated B16F0 cells (Figure S6). Besides PKA activity, the exchange protein directly activated by cAMP (EPAC) also transmits intracellular signals to regulate cAMP-dependent gene expression through the phosphorylation of CREB 40. EPAC inhibitor (ESI-09) decreases α-MSH- or db-cAMP-induced melanin production 41. These results suggest that signaling pathways through p38^MAPK^-MSK1 and cAMP-PKA axes could undertake different phosphorylation circuits on CREB and CRTC1 in response to α-MSH.

Treatment with p38^MAPK^ or MSK1 inhibitors consequently decreased cellular levels of MITF-M as well as melanin production in α-MSH-activated B16F0 cells, as did that with BI2B (Figure 8C and D). Moreover, the chemical inhibitors did not alter MTT assay-based viability of B16F0 cells (Figure S7), excluding from non-specific cytotoxicity. Overall, BI2B and chemical inhibitors of p38^MAPK^ or MSK1 could interrupt the same melanogenic signaling pathway in the expression of MITF-M in response to α-MSH.

### Suppression of α-MSH-induced expression of MITF-M-target melanogenic genes by BI2B

**Figure 9:**
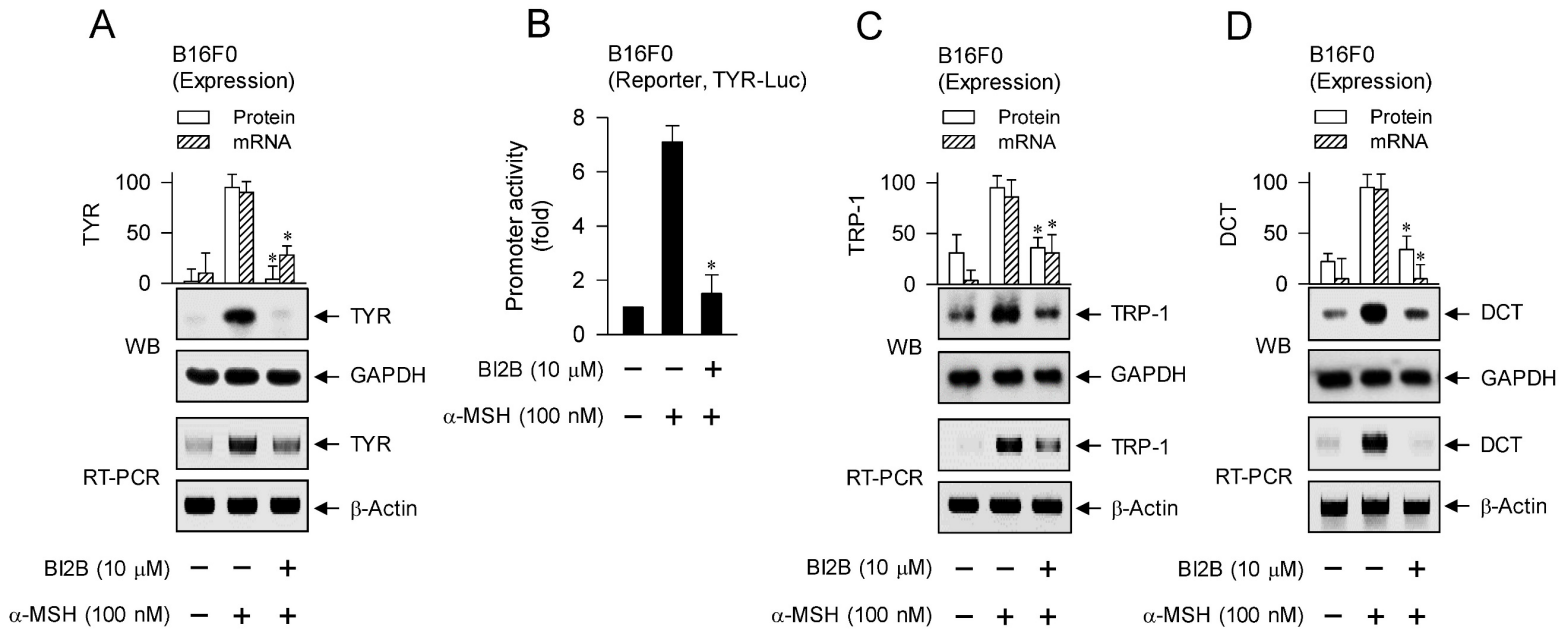

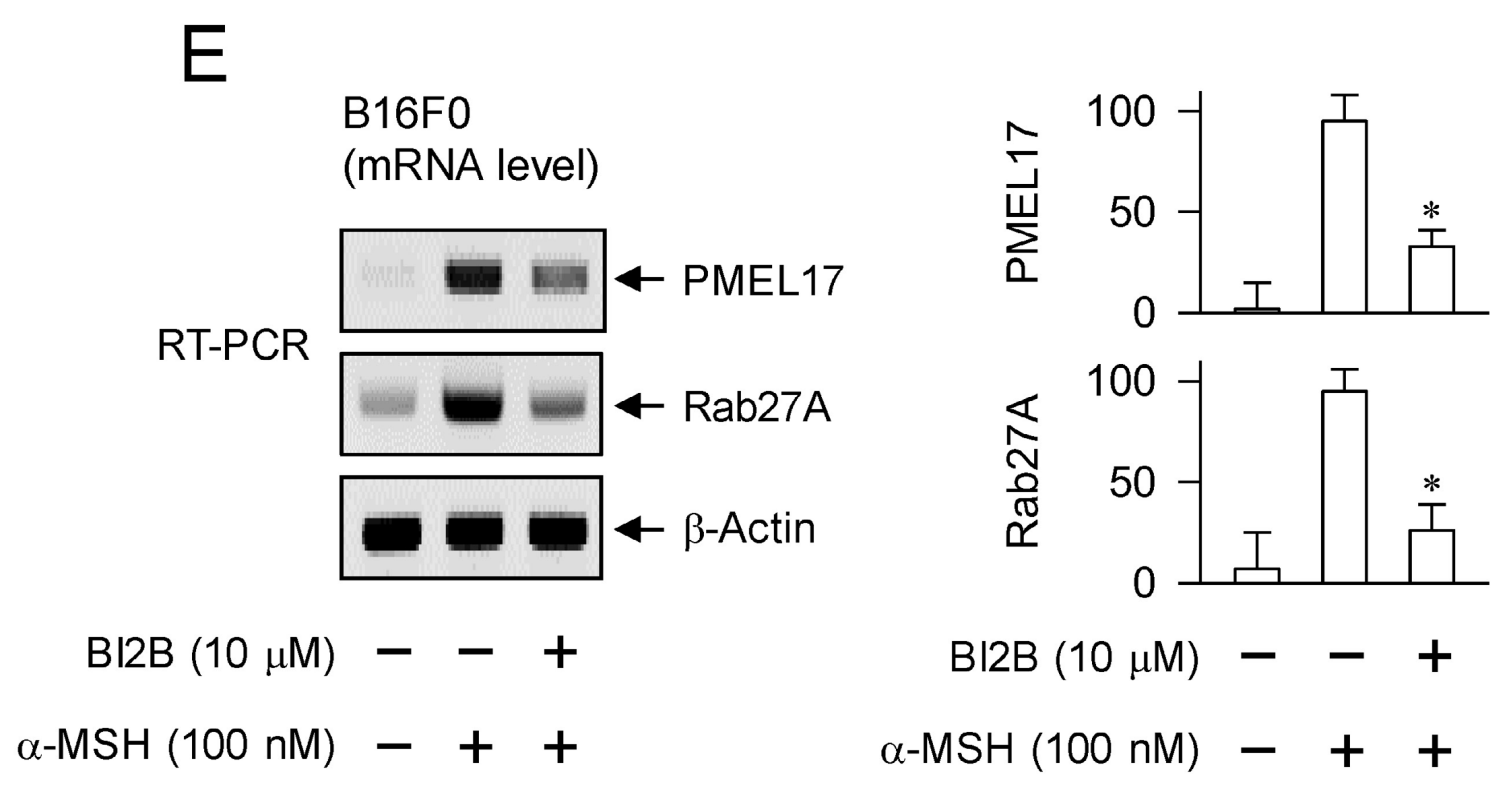
** Effect of BI2B on α-MSH-induced expression of MITF-M-target melanogenic genes.** B16F0 cells were stimulated with α-MSH in the presence of BI2B for 24 h (mRNA levels at A, C-E) or 48 h (protein levels at A, C, D). (A) BI2B suppressed α-MSH-induced expression of TYR. (B) B16F0 cells harboring TYR-Luc reporter were stimulated with α-MSH in the presence of BI2B for 20 h. BI2B inhibited α-MSH-induced promoter activity of TYR. (C, D) BI2B suppressed α-MSH-induced expression of TRP-1 and DCT. (E) BI2B suppressed α-MSH-induced transcription of PMEL17 and Rab27A. *P < 0.05 vs. α-MSH alone.

MITF-M up-regulates the expression of melanogenic genes encoding TYR, TRP-1 or DCT in melanin biosynthesis at melanosomes and PMEL17 or Rab27A in the transfer of pigmented melanosomes from melanocytes to overlaying keratinocytes in the skin 10, 28, 42. α-MSH-induced melanogenic programs up-regulated the expression of MITF-M in melanocyte cultures, which was suppressed by treatment with BI2B (Figure 3A and B). Subsequently, we examined that BI2B could affect MITF-M-dependent expression of melanogenic genes. B16F0 cells up-regulated the expression of TYR upon stimulation with α-MSH, which was suppressed by treatment with BI2B (Figure 9A). To clarify transcriptional regulation of TYR by BI2B, B16F0 cells were transfected with TYR-Luc, a plasmid construct containing the promoter region (-2236/+59) of TYR and the reporter, encoding firefly luciferase. B16F0 cells harboring the reporter plasmid increased firefly luciferase activity upon stimulation with α-MSH, indicating that α-MSH could stimulate the promoter activity of TYR (Figure 9B). Treatment with BI2B inhibited α-MSH-induced promoter activity of TYR (Figure 9B). These results suggest that BI2B could suppress α-MSH-induced expression of TYR at the promoter level. Notably, treatment with BI2B also suppressed the expression of TRP-1 or DCT (Figure 9C and D) as well as the transcription of PMEL17 or Rab27A in α-MSH-activated B16F0 cells (Figure 9E). Overall, treatment with BI2B could suppress the expression of MITF-M-target melanogenic genes.

## Discussion

**Figure 10:**
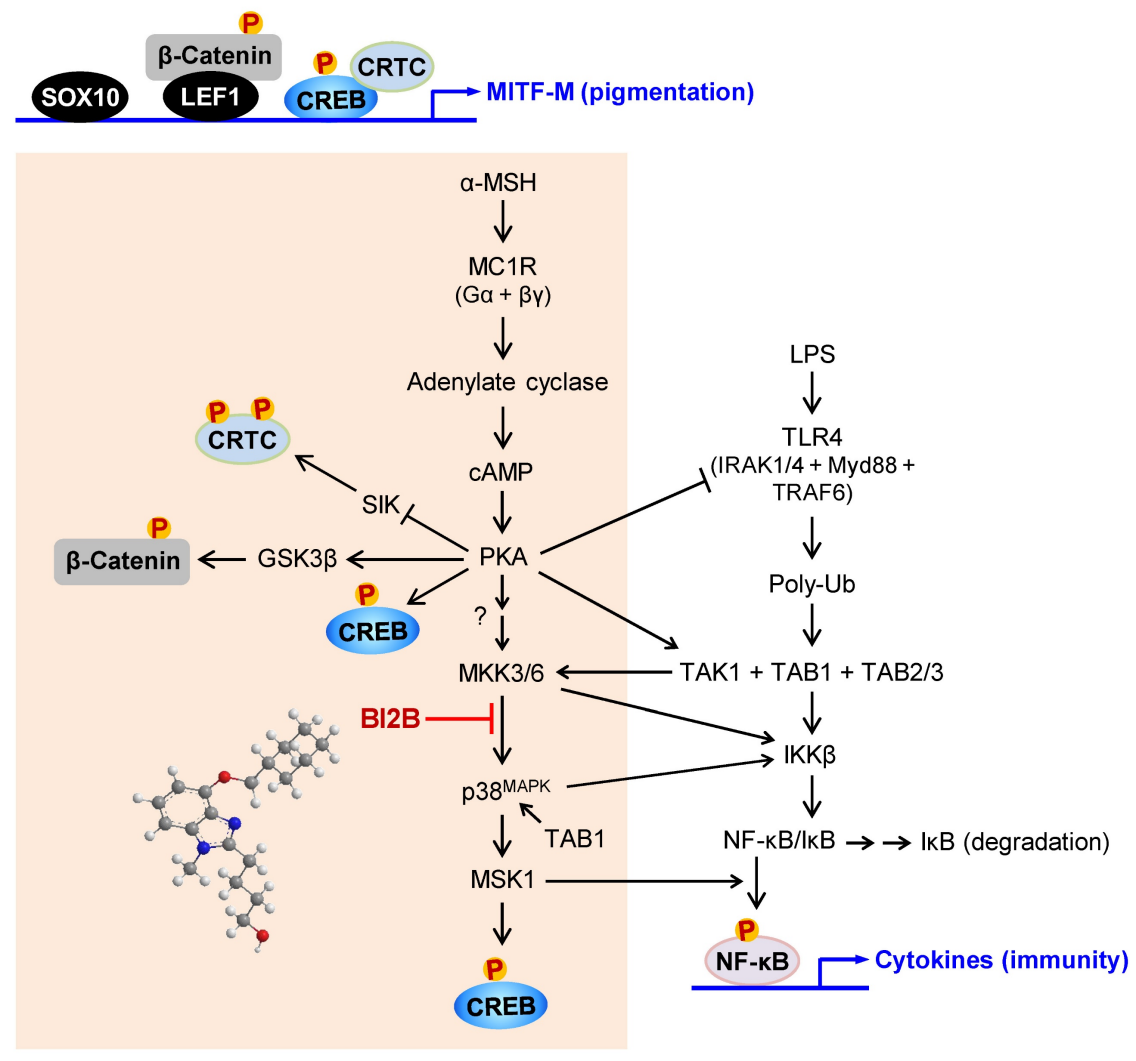
** A proposed mechanism of BI2B on antimelanogenic activity.** MC1R, a receptor of α-MSH, is exclusively present in melanocytes. BI2B suppressed the expression of MITF-M via decreasing the phosphorylation of CREB in α-MSH-induced melanogenic programs. As a molecular target, BI2B directly inhibited MKK3-catalyzed kinase activity on p38^MAPK^, and interrupted the downstream pathway of p38^MAPK^-MSK1-CREB in the expression of MITF-M through the phosphorylation of CREB. TLR4, a receptor of LPS, is distributed in immune and other cell types including melanocytes. LPS/TLR4 transmit intracellular signals to activate TAK1 activity, which is then branched to either MKK3/6 for MITF-M expression in pigmentation or IKKβ for NF-κB activation in immunity.

In the current study, we propose a molecular target of antimelanogenic efficacy by elucidating the inhibitory mechanism of BI2B on facultative melanogenesis. Topical treatment with BI2B ameliorated skin hyperpigmentation via UV-B-irradiated facultative melanogenesis in mice. BI2B also suppressed the protein or mRNA levels of melanogenic markers, such as MITF-M, TYR and POMC, in UV-B-exposed and pigmented skins. Moreover, BI2B inhibited melanin production in cell-culture models, such as UV-B-irradiated co-cultures of keratinocyte and melanocyte cells and α-MSH-activated melanocyte cultures. Mechanistically, BI2B inhibited the phosphorylation of CREB at the S133 residue in α-MSH-induced melanogenic programs, and suppressed the expression of MITF-M at the promoter. As shown in Figure 10, BI2B directly inhibited MKK3-catalyzed kinase activity on p38^MAPK^. Consequently, BI2B interrupted the downstream melanogenic pathway of p38^MAPK^-MSK1-CREB in α-MSH-induced expression of MITF-M. However, BI2B bypassed the axes of CRTC1, β-catenin and SOX10 in α-MSH-induced melanogenic programs.

BI2B inhibited the phosphorylation (activation) of p38^MAPK^ but not those of ERK and JNK in α-MSH-activated melanocyte cultures. Mammalian p38^MAPK^ family consists of four isoforms, such as p38α, β, γ, and σ 35. The p38α and β are ubiquitously expressed, whereas p38γ and σ are more tissue-specific, and cellular p38α are usually at higher levels than other isoforms 35. The p38^MAPK^ family is activated by almost all environmental stresses, including UV radiation and inflammatory stimuli, and integrates may types of intracellular signals, thus contributing to the biological responses 35. The p38^MAPK^ activity is tightly regulated, in which upstream kinases MKK3/6 directly phosphorylate p38^MAPK^ for activation 35, 43. MKK3 preferentially activates p38α and β, and MKK6 can phosphorylate all isoforms 35, 43. In another, the kinase activity of TAK1 phosphorylates MKK3/6, thus linking to the activation of p38^MAPK^ activity 35, 43. The p38^MAPK^ can be activated in MKK3/6-independent mechanism by directly interacting with TAB1, thus leading to the autophosphorylation (activation) of MKK3/6 44. Chemical inhibitors of p38^MAPK^ activity are clinically applied to the patients with inflammation and autoimmune disorders, such as early Alzheimer disease, multiple myeloma, cardiomyopathy and muscular dystrophy 45. Here, we propose that chemical inhibition of MKK3 activity in the axis with p38^MAPK^ could prevent acquired hyperpigmentation in the skin.

BI2B inhibits LPS/TLR4-mediated transcriptional activity of NF-κB 26, as well as melanin production in the current study. Melanogenic programs can cross-talk with signaling pathways of NF-κB activation through TLRs 24, as represented in Figure 10. LPS/TLR4 transmit intracellular signals to induce the autophosphorylation TAK1 at the T187 residue, which results in partial gain of TAK1 activity 46. PKA in melanogenic programs to regulate the expression of MITF-M can phosphorylate TAK1 at the S412 residue, thus fully activating the kinase activity of TAK1 47. TAK1 activity is then branched to MKK3/6 or IKKβ 46. Ten kinds of MAPKKKs are known to activate MKK3/6 35, 36. However, it remains to be defined which MAPKKKs like TAK1 are involved in α-MSH-induced melanogenic programs. IKKβ phosphorylates IκB, and p-IκB is degraded by ubiquitination-proteasome system 48. NF-κB freed from IκB can be translocated into the nucleus, where a heterodimer of NF-κB p50 and p65 binds to the *cis*-acting κB consensus on promoter regions 48. Nuclear MSK1 phosphorylates NF-κB p65 at the S276 residue in addition to the phosphorylation of CREB at the S133 residue, which facilitates the recruitment of CBP/p300 13, 31, 48. CBP/p300 can acetylate not only nucleosomal histones to become transcriptionally active in chromatin structure but also NF-κB p65 to enhance the transcriptional activity of NF-κB in another mechanism 13, 48.

## Conclusions

Taken together, we propose the interruption of MKK3-p38^MAPK^-MSK1-CREB pathway in the expression of MITF-M as a rationale to ameliorate hyperpigmentation via facultative melanogenesis and a potential strategy to treat acquired pigmentary disorders in the skin.

## Supplementary Material

Supplementary figures and table.
