# Supplementary material for: Interruption of p38MAPK-MSK1-CREB-MITF-M pathway to prevent hyperpigmentation in the skin
Source: Int J Biol Sci. 2024 Feb 17;20(5):1688–704. doi: 10.7150/ijbs.93120 (PMC10929196; doi:10.7150/ijbs.93120)
Supplement: Supplementary file 1 — Supplementary figures and table. [file ijbsv20p1688s1.pdf]

Supplementary Material

Title: Interruption of $p38^{MAPK}$-MSK1-CREB-MITF-M pathway to prevent hyperpigmentation in the skin

Authors: Song-Hee Kim,[1,*] Jiyeon Lee,[1,*] Jihye Jung,[1] Ga Hyun Kim,[1] Cheong-Yong Yun,[1] Sang-Hun Jung,[2] Bang Yeon Hwang,[1] Jin Tae Hong,[1] Sang-Bae Han,[1] Jae-Kyung Jung,[1,⊠] Youngsoo Kim[1,⊠]

Affiliations: [1]College of Pharmacy, Chungbuk National University, Cheongju 28160, Korea

[2]College of Pharmacy, Chungnam National University, Daejeon 34134, Korea

---

Contents

Table S1.

Figures S1 to S4.

Table S1. Nucleotide sequences of PCR primers.

Target		Nucleotide sequence	Amplicon
β-Catenin (B16F0)	Forward	5’-TGCAGATCTTGGACTGGAC-3’	134 bp
	Reverse	5’-CATGCTCCATCATAGGGTCCA-3’	
CREB (B16F0)	Forward	5'-AAGCTGAAAGTCAACAAATGACAGTT-3'	139 bp
	Reverse	5'-TGGACTGTCTGCCCATTGG-3’	
CRTC1 (B16F0)	Forward	5’-TCCCCAACATCATCCTCAC-3’	138 bp
	Reverse	5’-GGTCAATCTTCAGCTCGTC-3’	
DCT (B16F0)	Forward	5’-ACAGACGACTGGCTTGGAGCAGCAA-3’	517 bp
	Reverse	5’-ACATTCGGTTGTGACCAATGGGTGC-3’	
MITF-M (HEM)	Forward	5'-TCTACCGTCTCTCACTGGATTG-3'	142 bp
	Reverse	5'-GCTTTACCTGCTGCCGTTGG-3’	
MITF-M (B16F0)	Forward	5’-TACAGTCACTACCAGGTGCAG-3’	397 bp
	Reverse	5’-CCATCAAGCCCAAAATTTCTT-3’	
PMEL17 (B16F0)	Forward	5'-ATCAATGGGAGCCAGGTGTG-3'	479 bp
	Reverse	5'-AGGGTCCCAGTACCATCTCC-3’	
POMC (HRM2)	Forward	5'-CAACCTGCTGGCTTGCATC-3'	521 bp
	Reverse	5'-GGCTCTTCTCGGAGGTCATG-3’	
Rab27A (B16F0)	Forward	5'-GGGCAGGAGAGGTTTCGTAG-3'	508 bp
	Reverse	5'-CTTGGTCTCTACAGCGGAGC-3’	
SOX10 (B16F0)	Forward	5'-CAGACTGGAGGAGAGGTCGG-3'	122 bp
	Reverse	5'-GGTCTTGTTCCTCGGCCATG-3’	

TRP-1 (B16F0)	Forward	5'-GATATGGCGAAGCGCACAACTCACC-3'	536 bp
	Reverse	5'-AGACGCTGCACTGCTGGTCTCCCTA-3’	
TYR (B16F0)	Forward	5’-TACAGTCACTACCAGGTGCAG-3’	1211 bp
	Reverse	5’-CCATCAAGCCCAAAATTTCTT-3’	
β-Actin (HEM)	Forward	5'-GGACTTCGAGCAAGAGATGG-3'	234 bp
	Reverse	5'-AGCACTGTGTTGGCGTACAG-3‘	
β-Actin (HRM2, B16F0)	Forward	5’-TGGAATCCTGTGGCATCCATGAAAC-3’	349 bp
	Reverse	5’-TAAAACGCAGCTCAGTAACAGTCCG-3’	

Abbreviation: bp, base pairs.

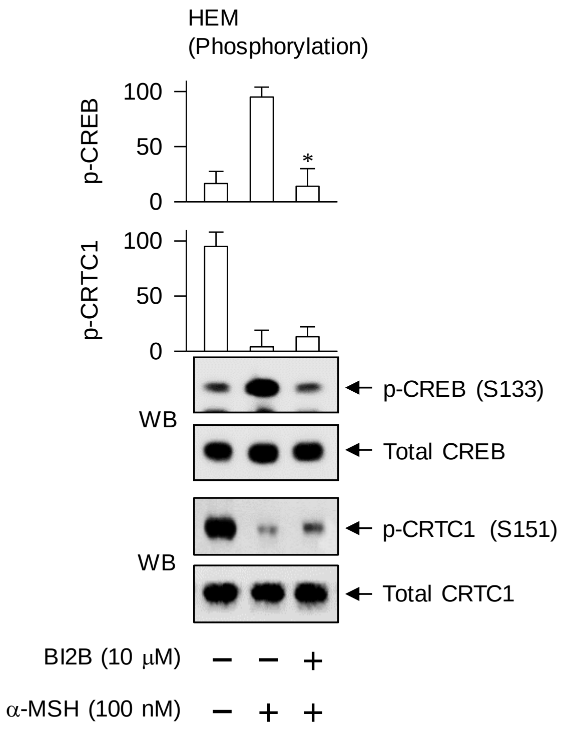

Figure S1. Effects of BI2B on CREB phosphorylation and CRTC1 dephosphorylation in α-MSH-activated HEM cells. The cells were pretreated with BI2B for 2 h and stimulated with α-MSH for 30 min in the presence of BI2B. Protein extracts were subjected to Western blot (WB) analysis. BI2B inhibited the phosphorylation of CREB but did not alter the dephosphorylation of CRTC1 in response to α-MSH. *$P < 0.05$ vs. α-MSH alone.

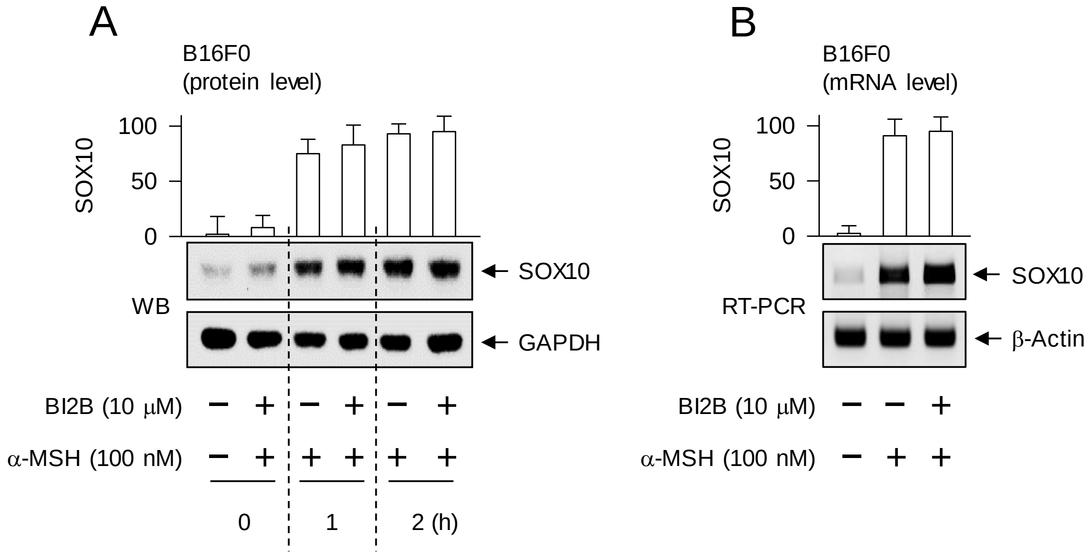

Figure S2. Effect of BI2B on SOX10 expression in α-MSH-activated B16F0 cells. The cells were pretreated with BI2B for 2 h and stimulated with α-MSH for indicated time points (A) or 1 h (B) in the presence of BI2B. Protein extracts were subjected to Western blot (WB) analysis (A) and total RNAs to RT-PCR analysis (B). BI2B did not alter α-MSH-induced protein and mRNA levels of SOX10.

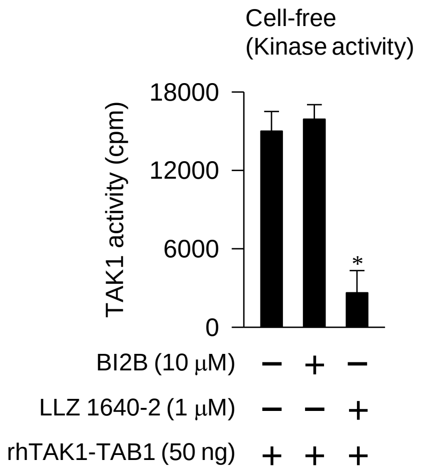

Figure S3. Effect of BI2B on the kinase activity of TAK1. Catalytically active rhTAK1-TAB1 was treated with BI2B for 10 min and its kinase activity was measured in cell-free reactions. BI2B did not inhibit TAK1-catalyzed kinase activity. LLZ 1640-2 was employed as a TAK1 inhibitor. $^*P < 0.05$ vs. rhTAK1-TAB1 alone.

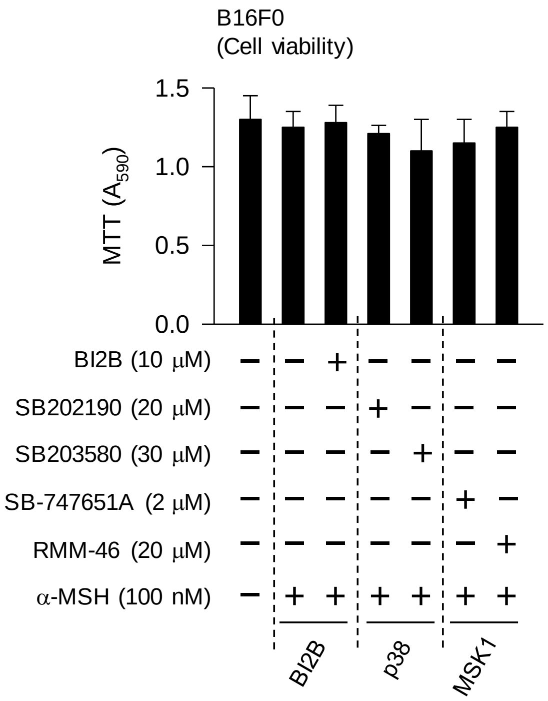

Figure S4. Effects of p38 and MSK1/2 inhibitors on cell viability. B16F0 cells were incubated with p38^{MAPK} inhibitors (SB202190 and SB203580) or MSK1 inhibitors (SB-747651A and RMM-46) in the presence of α-MSH for 72 h, and subjected to MTT assay. Both p38^{MAPK} and MSK1 inhibitors did not alter the viability of B16F0 cells.
